# Assignment of chromosomal locations for unassigned SNPs/scaffolds based on pair-wise linkage disequilibrium estimates

**DOI:** 10.1186/1471-2105-11-171

**Published:** 2010-04-07

**Authors:** Mehar S Khatkar, Matthew Hobbs, Markus Neuditschko, Johann Sölkner, Frank W Nicholas, Herman W Raadsma

**Affiliations:** 1 Reprogen - Animal Bioscience, Faculty of Veterinary Science, University of Sydney, Camden NSW 2570, Australia; 2 University of Natural Resources and Applied Life Sciences, Vienna, A-1180, Austria

## Abstract

**Background:**

Recent developments of high-density SNP chips across a number of species require accurate genetic maps. Despite rapid advances in genome sequence assembly and availability of a number of tools for creating genetic maps, the exact genome location for a number of SNPs from these SNP chips still remains unknown. We have developed a locus ordering procedure based on linkage disequilibrium (LODE) which provides estimation of the chromosomal positions of unaligned SNPs and scaffolds. It also provides an alternative means for verification of genetic maps. We exemplified LODE in cattle.

**Results:**

The utility of the LODE procedure was demonstrated using data from 1,943 bulls genotyped for 73,569 SNPs across three different SNP chips. First, the utility of the procedure was tested by analysing the masked positions of 1,500 randomly-chosen SNPs with known locations (50 from each chromosome), representing three classes of minor allele frequencies (MAF), namely >0.05, 0.01<MAF ≤ 0.05 and 0.001<MAF ≤ 0.01. The efficiency (percentage of masked SNPs that could be assigned a location) was 96.7%, 30.6% and 2.0%; with an accuracy (the percentage of SNPs assigned correctly) of 99.9%, 98.9% and 33.3% in the three classes of MAF, respectively. The average precision for placement of the SNPs was 914, 3,137 and 6,853 kb, respectively. Secondly, 4,688 of 5,314 SNPs unpositioned in the Btau4.0 assembly were positioned using the LODE procedure. Based on these results, the positions of 485 unordered scaffolds were determined. The procedure was also used to validate the genome positions of 53,068 SNPs placed on Btau4.0 bovine assembly, resulting in identification of problem areas in the assembly. Finally, the accuracy of the LODE procedure was independently validated by comparative mapping on the hg18 human assembly.

**Conclusion:**

The LODE procedure described in this study is an efficient and accurate method for positioning SNPs (MAF>0.05), for validating and checking the quality of a genome assembly, and offers a means for positioning of unordered scaffolds containing SNPs. The LODE procedure will be helpful in refining genome sequence assemblies, especially those being created from next-generation sequencing where high-throughput SNP discovery and genotyping platforms are integrated components of genome analysis.

## Background

The last decade has seen a rapid expansion in the number of genomes from a diverse range of species being sequenced [1]. Further developments of high-throughput sequencing platforms are likely to accelerate the sequencing of potentially many more genomes [2]. Furthermore, such data sets may be coupled with high-throughput SNP-analysis platforms to undertake population diversity characterization [3,4]. The relatively short sequence reads from the high-throughput systems pose challenges in the creation and ordering of contigs and scaffolds in the absence of a mature reference genome. Ordering closely linked markers is also a challenge using linkage mapping. Assembly of the bovine genome sequence has recently been reported [5]. In the course of bovine sequencing to date, more than 2 million SNPs have been discovered and more SNPs are being added with additional sequencing efforts using next generation sequencing technologies [6], resulting in several high-density SNP-genotyping platforms for population-wide screening of genome diversity. Despite several genome builds, there are still a large number of scaffolds and SNPs that are not yet assigned to any chromosomes. For example, there are 11,869 un-ordered scaffolds in Btau4.0, constituting 9.72% (263.4 Mb) of the bovine genome. In order to improve the genome assembly, it would be useful to assign un-ordered scaffolds and SNPs to chromosomes, and to locations within chromosomes [7].

A number of strategies can be adopted to place polymorphic markers on chromosomes via linkage maps [8-10], Radiation Hybrid maps [11-13], FISH and integrated maps [14]. Linkage studies require genotypic information on specific families, and it is difficult to construct accurate or high-resolution linkage maps for high-density SNP data [10]. Alternatively, physical maps of SNPs, created by screening RH panels, enable high-resolution positioning of SNPs but require high-density anchoring of the physical genome to the assembly. However, a SNP can be given a chromosomal position based on linkage disequilibrium (LD) information of the SNP with other SNPs with known position in the genome. LD analysis does not rely on family information and decays rapidly across ([15], and within populations [16]) and, as such, can provide a means to accurately position SNPs based on LD relationships with other SNPs with known map positions. Miller *et al*., [17] applied an LD-based approach to map a test set of SNPs with known map positions. However, the utility of this approach for unmapped SNPs, or SNPs with ambiguous positions in the context of high-density SNP data, has not been demonstrated.

Recently we showed [18] that polymorphic markers can be ordered within a chromosome based on pair-wise LD only and termed this procedure LODE (Locus Ordering by Dis-Equilibrium). A sorting algorithm (sorting points into neighbourhoods) [19] was applied. The procedure was successful in assigning a small number of unmapped SNPs to unique chromosomal locations but was found to be limited in terms scaling up to large matrices representing dense SNP panels.

Here we modify the initial LODE procedure for assigning SNPs to chromosomes and positioning SNPs within chromosomes. First, the efficiency of using genome-wide LD information is investigated by using mapped SNPs as a test set. Next, the procedure is applied to assign positions for 4,688 out of 5,314 unpositioned SNPs on Btau4.0, which were either un-assigned or assigned with ambiguity based on BLAST against Btau4.0, from a high-density SNP panel of 73,568 SNPs. We also suggest the chromosomal locations of un-ordered scaffolds. Finally the LODE procedure was used to confirm the order of mapped SNPs across the genome as a means to check the quality of genome assembly.

## Methods

### Genotypic Data

Data from three SNP genotyping arrays, namely 15 k [20], 25 k (Affymetrix; http://www.affymetrix.com) and 54 k (Illumina; http://www.illumina.com/), used for genotyping 1,536, 441 and 377 Australian Holstein-Friesian (HF) bulls, respectively, were combined into a single dataset for the current analyses. There were duplicate samples and duplicate SNPs within and between datasets. Only unique samples and SNPs with higher call rate (% genotype assignment) were selected to include in the final dataset. Any inconsistent genotype was set to unknown. The final combined dataset represented 73,569 unique SNPs and 1,943 bulls with an average of 628 bulls genotyped per SNP. The mean coefficient of coancestry among these 1,943 bulls is 0.025, with 0.0 and 0.035 for the first and third quartiles, respectively.

### Position of SNPs

The location of each of the 73,569 SNPs in the bovine genome was assessed from BLAST alignment of SNP flanking sequences with the Btau4.0 assembly ftp://ftp.hgsc.bcm.tmc.edu/pub/data/Btaurus/fasta/Btau20070913-freeze/, which includes a considerable quantity of sequence (organised as either a set of scaffolds or as a pseudo-chromosome) that is not assigned to a chromosome (referred to as 'Un'). We used the Batu4.0 assembly to demonstrate utility of the LODE procedure since the assembly contained a number of SNPs not assigned to chromosomes. Comparison of LODE positions were also made against another bovine assembly build UMD3.0 which has recently become available.

SNP positions on Btau4.0 were categorised as follows: i) 'mapped' (single assignment to a chromosome); ii) 'ambiguous' (more than one assignment in the genome); iii) 'Un' (single assignment to 'Un' sequences only); iv) 'unassigned' (no assignments in the genome). Collectively, the last three categories (ambiguous, Un and unassigned) are here called 'unpositioned'.

### LODE procedure

The location of each unpositioned SNP was estimated on the basis of its LD (estimated as *r*^2^) with mapped SNPs. The *r*^2 ^estimates were obtained using GOLD [21]. The genotypes for SNPs on the X-chromosome were considered as homozygous for the purpose of computing LD estimates. Only high quality LD estimates (significant at the 0.01 level, and estimated from a minimum of 100 observations) were used. The actual procedure used in the present study is an extension of the strategy first used by Miller *et al*. [17] and subsequently adapted to the LODE procedure by Sölkner *et al*. [18]. In the present study, the LODE procedure consisted of two main steps: A) assigning a SNP to a chromosome; B) estimating the position of the SNP within the assigned chromosome. After trialling many combinations of criteria, the following strategy was used. (The relative accuracy of using different threshold combinations is shown in Additional file 1).

#### A) Assigning a SNP to a chromosome

For each unpositioned SNP with MAF >0.01:

1. *r*^2 ^was estimated with all mapped SNPs.

2. From these estimates of *r*^2^, two parameters were computed with respect to each chromosome, namely:

a. maximum *r*^2 ^(*r*^2^_*max*_, as an indicator of the strength of LD)

b. number of mapped SNPs with *r*^2 ^> 0.1 (*n*_0.1_, as an indicator of the number of mapped SNPs in LD with the unpositioned SNP)

3. Chromosomes were then ranked according to *r*^2^_*max *_and *n*_0.1_, in the latter case after excluding chromosomes for which *n*_0.1_<3. A chromosome with top ranking for both parameters was identified as the candidate chromosome for that unpositioned SNP.

After trialling the above threshold combinations, SNP with MAF ≤ 0.05 required an additional check to improve accuracy of placement. In addition to the above strategy (steps 1-3), the chromosome with next highest *r*^2^_*max *_was identified. If the *r*^2^_*max *_of the second chromosome exceed 2/3 *r*^2^_*max *_of the candidate chromosome, the SNP was not assigned to any chromosome. This improved the accuracy of assignment from 92.1% to 98.9% (Additional file 1). SNPs which didn't meet these criteria were left unpositioned.

#### B) Estimating position within an assigned chromosome

For each unpositioned SNP that could be assigned to a chromosome, its location on that chromosome was allocated the same position as that of the mapped SNP with which the unpositioned SNP has *r*^2^_*max*_.

The above LODE procedure was first tested for its ability to determine the location of SNPs whose location was actually known. Three test sets involved determining the location of a total of 1,500 "masked" SNPs (50 from each of the 29 autosomes and the X chromosome, randomly selected from SNPs with known positions). Each set comprised SNPs with a different MAF class, namely 0.001<MAF ≤ 0.01 (300 SNPs, 10 from each chromosome); 0.01<MAF ≤ 0.05 (300 SNPs, 10 from each chromosome); >0.05 (900 SNPs, 30 from each chromosome). The extent to which the procedure was successful was assessed in terms of "efficiency" (the percentage of "masked" SNPs that were assigned a location), "accuracy" (the percentage of "masked" SNPs that were assigned to the correct chromosome), and "precision" (the difference in physical distance between the known position and the assigned position). After testing the LODE procedure with the above test sets, the same procedure was applied to unpositioned bovine SNPs.

### Comparative position on human genome

To provide further evidence of the utility of the LODE procedure, we used a comparative mapping approach to confirm the genome location of unpositioned bovine SNPs against the human genome assembly hg18, since this represents the most complete mammalian genome to date. This approach was considered helpful since the location of the unpositioned SNPs could not be validated on Btau4.0 directly.

The comparative position of bovine SNPs was estimated in the human genome using two approaches. Firstly, BLAST was used to align the flanking sequences of unpositioned SNPs with the hg18 assembly ftp://hgdownload.cse.ucsc.edu//goldenPath/hg18/. Secondly, the 'LiftOver' tool http://genome.ucsc.edu/cgi-bin/hgLiftOver was used with default settings to convert LODE positions from the bovine Btau4.0 assembly to the human hg18 assembly.

### LODE as a means for checking genome assembly

The LODE procedure was used to recompute the positions of all SNPs mapped to the genome which were genotyped and met minimum criteria for inclusion as detailed above and MAF >0.05. The procedure was performed in batches, where the positions of 10% (every 10th) of SNPs of a chromosome were masked. The positions of the masked SNPs were recomputed based on the LD information of the remaining SNPs in the genome. The chromosomal assignments and positions estimated by LODE were compared with original positions on Btau4.0 and also with UMD3.0.

## Results

### Validation of LODE procedure by test runs

A total of 870 (96.7%) of the 900 test SNPs with MAF>0.05 were allocated a chromosomal position by LODE. All but one (i.e. 869 = 99.9%) of the positions were the same as the Btau4.0 accepted assembly position. The comparison of estimated and known SNP positions (Additional file 2) shows strong agreement (mean Pearson's correlation = 0.98 across all chromosomes). The mean precision of localisation was 914 ± 130 kb (Table 1). The results from alternate criteria that were tested during the development of the preferred strategy are shown in Additional file 1.

**Table 1:** Efficiency (proportion of SNPs placed), accuracy (proportion of SNPs placed correctly) and precision (kb location from draft assembly location) of the LODE procedure for placing SNPs with known location in three test runs with varying thresholds of MAF of SNPs to be placed.

Test Run	Number of SNPs	Efficiency (%)	Accuracy (%)	Precision (kb)
Run1 (SNPs with MAF>0.05)	900	96.7	99.9	914 ± 130

Run2 (SNPs with 0.01<MAF ≤ 0.05)	300	30.6	98.9	3137 ± 381

Run3 (SNPs with 0.001<MAF ≤ 0.01)	300	2.0	33.3	6853 ± NA

92 SNPs (30.6%) from the second test set (0.01<MAF ≤ 0.05) were positioned, with only one mis-assignment (1.1%). Comparison of the estimated LODE positions and the known positions showed high agreement (Additional file 3). Thus, the efficiency of positioning SNPs in this MAF range was much lower, but for those SNPs that could be positioned, the accuracy was very high. Rare SNPs (0.001 *<*MAF ≤ *0.01) *could not be positioned (Table 1). Overall, it can be concluded that the LODE procedure can position SNPs with MAF>0.01 with high accuracy.

### Application of LODE to unpositioned SNPs

In the Btau4.0 assembly, there are 6,470 'unpositioned' SNPs. Of these, 5,314 SNPs have MAF>0.01, making them suitable for LODE mapping (Additional file 4).

Table 2 shows the number of SNPs positioned by LODE. Of the 5,314 'unpositioned' SNPs with MAF >0.01, 2,291 had ambiguous positions, 1770 were aligned to 'Un' sequences, and 1,253 were unaligned. Using the LODE strategy, 4,688 of the 'unpositioned' SNPs were positioned. Of the 626 SNPs which didn't meet the thresholds of the LODE procedure, 231 had ambiguous positions, 271 had 'Un' sequences and 124 were unaligned. As expected from the test-set results, a higher proportion of the SNPs with MAF >0.05 (94.2%) than with 0.01<MAF ≤ 0.05 (27.6%) could be positioned. The proportions of SNPs placed in the two categories are comparable to the proportions observed in the two corresponding test sets.

**Table 2:** Number of 'unpositioned' SNPs assigned to specific chromosomes by LODE.

	ambiguous (multi-hits against Btau4.0)	'Un' (assignment to 'Un' sequence)	unaligned (no hit against Btau4.0)	Total 'unpositioned'
SNPs (>0.05 MAF) positioned by LODE	2006/2082 (96.3)	1466/1625 (90.2)	1085/1132 (95.8)	4557/4839 (94.2)

SNPs (0.01<MAF ≤ 0.05) positioned by LODE	54/209 (25.8)	33/145 (22.8)	44/121 (36.4)	131/475 (27.6)

Total SNPs positioned by LODE	2060/2291 (89.9)	1499/1770 (84.7)	1129/1253 (90.1)	4688/5314 (88.2)

All 'unpositioned' SNPs	2291	1770	1253	5314

Figures in parenthesis are per cent.

Of 2,291 SNPs in the ambiguous category, 2,060 were positioned by LODE. The SNPs in this category had multiple hits when flanking SNP sequence was BLASTed against Btau4.0. Although it is possible that some of the sequence alignment positions in this category may be the result of errors in the Btau4.0 assembly, it is more likely that they are genuine genomic positions reflecting structural polymorphisms or segmental duplications. The SNP positions estimated by LODE are approximations and hence for the SNPs in this category it may be preferable to use LODE positions to discriminate between the multiple sequence-alignment results, and use the sequence alignment consistent with LODE for final positioning.

Of 1,770 SNPs belonging to 'Un' sequences, 1,499 were positioned by LODE. These SNPs belong to 494 unique "Un" unordered Btau4.0 scaffolds. Assignment of these SNPs to definite chromosomes suggests the assignment and positions of respective "Un" scaffolds to the same chromosome as well. Table 3 presents the number and length of these "Un" scaffolds assigned to different candidate chromosomes. These assigned scaffolds comprise 87.7 Mb of genome sequence in total. There were multiple SNPs on some of the "Un" scaffolds. Out of these, 210 "Un" scaffolds had two or more SNPs (mean = 5.04) with all the SNPs aligned to one chromosome (Additional file 5). These 210 "Un" scaffolds with multiple SNPs could be assigned and some of them could be oriented on the chromosome, based on the SNP position estimates. This approach may therefore be very useful for improving the bovine assembly, since it provides for a higher resolution assignment of SNPs and the scaffolds.

**Table 3:** Number of SNPs and unassigned scaffolds ("Un") assigned to different chromosomes by the LODE procedure.

Chromosome	No. of SNPs assigned	No. of "Un" scaffolds assigned	Length of "Un" scaffolds in bp
1	290	31	3632458

2	213	12	1399643

3	219	13	2375137

4	176	15	1177590

5	198	9	2050871

6	235	18	3492179

7	212	17	2416408

8	197	22	3299957

9	183	18	2257082

10	149	16	2017247

11	152	17	1655602

12	215	16	4401081

13	152	13	2066119

14	228	12	4702182

15	139	18	3049502

16	212	24	5697862

17	117	11	845416

18	95	11	1530906

19	97	11	1769762

20	75	7	559988

21	115	20	2921283

22	87	10	1083985

23	84	5	829315

24	86	6	607772

25	62	5	398973

26	121	21	2117068

27	66	6	392536

28	94	11	1571243

29	126	12	1981261

X	293	78	25445183

Total	4688	485	87745611

There were 9 scaffolds with multiple SNPs that were given positions on two chromosomes by LODE. This may indicate problems in the assembly of these scaffolds themselves and may require the segments with separate SNPs to be placed separately for improved accuracy of genome assembly.

Of 1,253 SNPs in the unaligned category, 1,129 were positioned by LODE. These sequences are missing from the Btau4.0 assembly, possibly because of the nature of whole-genome shotgun sequencing, or because they are within polymorphic regions not present in the two individuals which contributed to Btau4.0, but are present within the population with which we have worked.

In summary, the LODE procedure has positioned 4,688 of 5,314 SNPs that are unpositioned in the Btau4.0 assembly.

### Validation of LODE positions by comparative mapping

**Figure 1:**
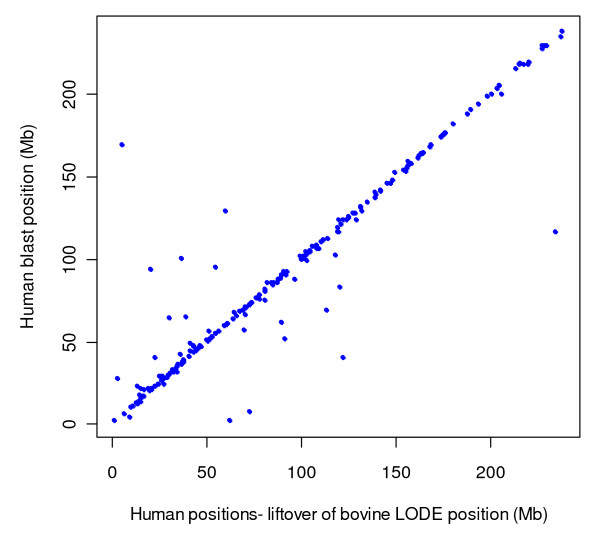
**Comparison of SNP positions on human assembly obtained with BLAST of flanking sequence of SNPs vs the positions obtained by 'LiftOver' of bovine LODE positions**. The comparisons for positions are shown in a single figure for the 230 SNPs combined across all chromosomes.

Unique (single location) positions on the human hg18 assembly were obtained from the BLAST and LiftOver procedures for 284 SNPs from the panel of 4,688 SNPs positioned by LODE. The chromosomal assignments for 230 (81%) of these SNPs were identical between BLAST and LiftOver. 54 (19%) of the 284 SNPs had different chromosomal assignments on hg18 by the two above procedures, which may be due to the LODE positions being outside of conserved syntenic blocks between bovine and human chromosomes. Such blocks are normally very small and quite variable in length. Comparison of the chromosomal positions of the 230 SNPs, with same chromosomal assignments, shows very strong agreement (cor = 0.95) (Figure 1) between the positions obtained through BLAST and LiftOver. These results support the accuracy and utility of the LODE procedure for positioning SNPs with MAF>0.01.

### Checking genome assembly with LODE

Table 4 shows comparison of the chromosomal assignments of SNPs repositioned by LODE. Out of 54,062 SNPs tested 53,068 (98.16%) of the SNPs could be given a chromosomal assignment by LODE confirming the high efficiency seen in the pilot test batch of 1,500 SNPs. Most of the SNPs (99.9%) were given the same chromosomal assignments which indicate in general a high level of integrity of Btau4.0. A total of 81 SNPs were found to have different chromosomal assignments by LODE compared with Btau4.0.

**Table 4:** Chromosome-wise summary of repositioning done by LODE.

Chromosome	No. of SNPs tested	No. of SNPs assigned to same chromosome	No. of SNPs assigned a different chromosome	No. of SNPs not assigned
1	3635	3585	2	48

2	2774	2732	3	39

3	2612	2551	12	49

4	2517	2470	4	43

5	2260	2221	4	35

6	2597	2570	1	26

7	2244	2204	4	36

8	2378	2344	2	32

9	1993	1958	4	31

10	2281	2241	10	30

11	2389	2337	2	50

12	1677	1646	3	28

13	1909	1880	3	26

14	1771	1747	1	23

15	1808	1781	2	25

16	1654	1627	0	27

17	1661	1626	0	35

18	1474	1436	2	36

19	1577	1538	2	37

20	1616	1603	0	13

21	1377	1343	2	32

22	1313	1279	2	32

23	1258	1223	2	33

24	1339	1319	1	19

25	1087	1065	1	21

26	1062	1042	2	18

27	1014	969	1	44

28	1004	980	1	23

29	1070	1040	0	30

X	711	630	8	73

**Total**	**54062**	**52987**	**81**	**994**

**Figure 2:**
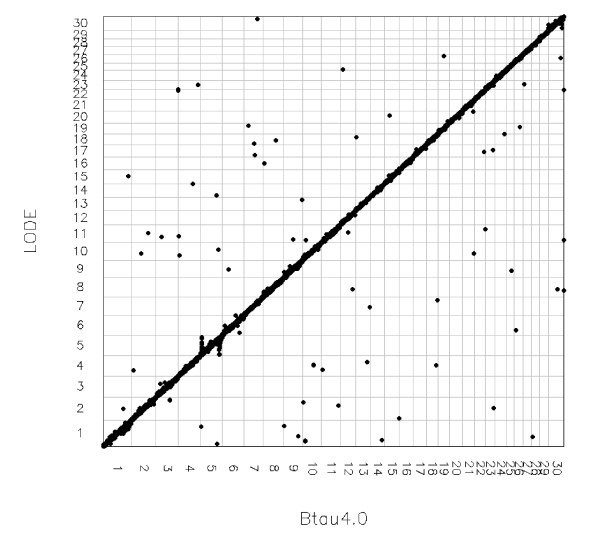
**Comparison of repositioned locations of SNP by LODE with original location on Btau4.0**. X-chromosome is labelled as 30.

**Figure 3:**
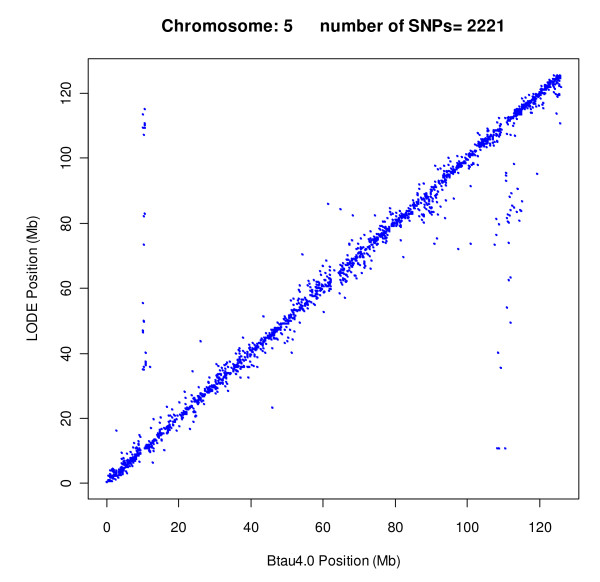
**The comparison of chromosomal assignments of 1776 SNPs repositioned by LODE procedure with original positions on Btau4.0 on chromosome 5**. Two potential problem regions (10-11 Mb and 90-120 Mb) on Btau4.0 on this chromosome can be noted.

Table 4 shows distribution of these 81 SNPs mapped to different chromosomes. Out of these SNPs, 5 blocks can be noted as shown in Table 5. All the SNPs of these blocks were assigned to a different chromosome by LODE. The positions of these SNPs were compared with another recently released assembly of the bovine genome (UMD3.0) which agrees with LODE assignments for the SNPs in the blocks (Table 5). These blocks suggest problem areas within the Btau4.0 assembly. The comparison of the overall agreement between LODE and SNPs positioned on Btau4.0 are shown in Figure 2 by the way of Oxford grid. The detailed alignment of LODE positions and Btau4.0 for each chromosome is shown in Additional file 6. This identifies the chromosomal regions which may suggest potential problem areas in the Btau4.0 assembly. In particular two regions (10-11 Mb and 90-120 Mb) on BTA5 suggest problem areas in the assembly of this chromosome (Figure 3). Similarly X-chromosome shows several regions where a relatively higher number of SNPs show differences in original Btau4.0 positions and LODE positions which may suggest general problem in the assembly of X-chromosome (Additional file 6).

**Table 5:** List of SNPs assigned a different chromosome by LODE as compared to Btau4.0 and 5 problem regions in Btau4.0 identified by LODE.

	Btau4.0		LODE		UMD3.0	
**SNP Assay ID**	**Chrom** **osome**	**Position** **(bp)**	**Chrom** **osome**	**Position** **(bp)**	**Chromosome: Position (bp)**	**Problem region**

ARS-BFGL-NGS-26766	1	114010038	2	69089678	2:71023597	

1861431	1	144225095	15	47262164	1:142952903	

BTA-45006-no-rs	2	10760381	4	34049395	4:33987224	

1876171	2	56762771	10	43036781	2:54560749	

1873289	2	94496897	11	59283090	11:61135811	

ARS-BFGL-NGS-32344	3	30329145	11	36536145	3:27963904	

**ARS-BFGL-NGS-115797**	**3**	**77488731**	**2**	**126296914**	**2:118277989**	**1**

**343487**	**3**	**77719948**	**2**	**125113698**	**2:118048961**	**1**

**351668**	**3**	**77757827**	**2**	**120653554**	**2:118011142**	**1**

**ARS-BFGL-NGS-89183**	**3**	**77786834**	**2**	**125153297**	**2:117982134**	**1**

**ARS-BFGL-NGS-317**	**3**	**77822712**	**2**	**123366377**	**2:117946210**	**1**

**1871424**	**3**	**77859105**	**2**	**121859331**	**2:117909817**	**1**

**ARS-BFGL-NGS-70541**	**3**	**127719186**	**22**	**60528302**	**22:60785491**	**2**

**1870128**	**3**	**127750012**	**22**	**57739278**	**22:60755275**	**2**

**ARS-BFGL-NGS-58676**	**3**	**127769201**	**22**	**61298196**	**22:60736089**	**2**

**ARS-BFGL-NGS-2807**	**3**	**127874807**	**22**	**54335047**	**22:60634400**	**2**

**ARS-BFGL-NGS-19124**	**3**	**127908628**	**22**	**61358781**	**22:60600490**	**2**

ARS-BFGL-NGS-8807	4	2607777	11	40976163	4:2676685	

ARS-BFGL-NGS-113798	4	4445195	10	30252857	4:4340312	

ARS-BFGL-NGS-15424	4	83768798	14	78612579	14:81878266	

344491	4	110028200	23	28037374	4:106890553//8:91314567	

1867566	5	5331062	1	120905947	5:5049476	

1868001	5	94598147	14	11525489	5:88617706	

BTA-110801-no-rs	5	96742247	1	16408220	1:16094228	

BTA-74608-no-rs	5	102914139	10	66434699	5:96344375	

464972	6	36820192	9	49534891	6:37434546	

Hapmap38285-BTA-110077	7	25557308	19	51160026	7:27891275	

1867647	7	60836776	18	6943970	7:63367359	

BTB-00316147	7	62064599	17	13362033	17:12137554	

BTA-98858-no-rs	7	77340598	X	69412698	X:121600055	

1873146	8	7400266	16	38314751	8:7357495	

464622	8	70460363	18	26000936	11:9122777 //18:24282247 //8:67661725	

BTA-97336-no-rs	9	3823907	1	125108946	9:4581947	

1865493	9	54935966	11	22728050	9:53194372	

BTB-01806856	9	81376121	1	61983636	1:62607089	

1866405	9	103328739	13	68455077	9:100810058	

ARS-BFGL-NGS-92893	10	3905779	2	110967662	10:4340696	

**ARS-BFGL-NGS-73149**	**10**	**15513722**	**1**	**35290138**	**1:30259943**	**3**

**BTA-97531-no-rs**	**10**	**15578191**	**1**	**32951433**	**1:30333360**	**3**

**BTB-01490270**	**10**	**15659183**	**1**	**31342947**	**1:30414871**	**3**

1874946	10	17857141	11	18153873	10:17528963	

**BTA-88378-no-rs**	**10**	**62466743**	**4**	**71372575**	**4:71413010**	**4**

**Hapmap28362-BTA-48086**	**10**	**62487185**	**4**	**68238697**	**4:71433452**	**4**

**ARS-BFGL-NGS-53167**	**10**	**62712521**	**4**	**69590750**	**4:71603530**	**4**

**BTA-109199-no-rs**	**10**	**62734149**	**4**	**71457638**	**4:71625158**	**4**

**1875511**	**10**	**62741080**	**4**	**72626961**	**4:71632089**	**4**

**ARS-BFGL-BAC-6642**	**11**	**7361519**	**4**	**37715495**	**4:37109034**	**4**

1861989	11	98919723	2	90294746	11:95497623	

ARS-BFGL-BAC-5532	12	14460102	25	1759284	12:15518882 //25:1416859	

349300	12	41051742	11	64226960	11:65590436	

1874001	12	69506929	8	47841259	12:72414147	

BTB-01588251	13	3055670	18	45334043	18:45474850	

344445	13	65183788	4	85581292	13:65259657	

464345	13	82520352	7	50231706	13:82162818	

ARS-BFGL-NGS-31537	14	64995274	1	41315505	14:69103642	

1875855	15	29419479	20	46515729	15:31389253	

BTA-113745-no-rs	15	81726795	2	12901670	2:15780878	

352984	18	57222022	4	66139068	18:57677863	

345202	18	64492276	7	93294065	18:64408080	

ARS-BFGL-NGS-77278	19	34916761	26	41917320	26:46541332	

ARS-BFGL-NGS-67396	19	55603678	20	10016943	19:54645063	

1859467	21	60422341	20	71596996	21:61747881	

ARS-BFGL-NGS-9612	21	64787627	10	40396512	21:66220013	

461240	22	54344094	17	31319897	22:53552948	

463748	22	60828419	11	84942870	22:59688782	

465954	23	42051287	17	42535225	23:41166590	

1860554	23	44428021	2	74386823	23:43645744	

BTA-58638-no-rs	24	55379446	18	64455859	24:53790841	

ARS-BFGL-NGS-55374	25	28795159	9	43457907	9:42792078	

1860594	26	10984398	6	31957704	26:10567934	

ARS-BFGL-NGS-22409	26	32419852	19	41655298	19:43295532	

ARS-BFGL-NGS-102734	27	6298951	23	31511369	23:25899622 //23:26148979	

ARS-BFGL-NGS-40170	28	5149426	1	58990404	28:6888276	

**1864508**	**X**	**48918943**	**8**	**45769443**	**8:42969739**	**5**

**ARS-BFGL-NGS-109695**	**X**	**48952760**	**8**	**46295084**	**8:43003529**	**5**

**1862184**	**X**	**48965834**	**8**	**46679390**	**8:43016396**	**5**

**BTB-01044512**	**X**	**48988556**	**8**	**45367300**	**8:43039113**	**5**

BTB-01631465	X	67394831	26	30689907	26:32336734	

1862196	X	87059858	22	57016127	X:146942067	

1863291	X	87885269	8	37568243	X:138709703	

ARS-BFGL-NGS-10360	X	88338874	11	17475323	X:137429436	

## Discussion

In this study we reported and validated a procedure to accurately and efficiently map SNPs based on LD information. The LODE procedure offers particular advantages in the positioning of problem SNPs for which no unambiguous assignment on a draft genome assembly could be made, as well as a means for positioning of unordered scaffolds containing SNPs. Miller *et al*. [17] used a genetic algorithm based approach and linkage disequilibrium to position a test set of bovine SNPs with known location, and applied a minimum threshold of *r*^2 ^>0.4 between SNPs in their method. Application of such a threshold would have resulted in lower efficiency (71% for SNPs in test Run1 (MAF >0.05) and slightly lower accuracy (2 mis-assignments) when compared to the thresholds adopted in our study (Table 1). The LODE procedure showed greater utility over the methods described by [17] where the authors have not demonstrated the placement of SNPs with MAF<0.05, SNPs with ambiguous assignments or unpositioned SNPs. The original LODE procedure of Solkner *et al*. [18] was of similar accuracy and efficiency in small test runs, but has severe limitations in terms of computing time (Solkner *et al*. in preparation) imposed by matrix dimensions of marker density, thus limiting application to full genome analyses. MAF of SNP to be placed has a significant effect on the efficiency of the LODE procedure, as shown in detail in the result section by running the three different test sets of varying MAF (Table 1). However despite the lower efficiency, the accuracy of the LODE procedure for SNPs with a 0.01<MAF ≤ 0.05 was high. Another advantage of using the LODE procedure was that SNPs which showed deviation from HWE could also be mapped. For example in the test set1, out the 56 SNPs showing HWE deviation (*P < 0.0001*), 54 could be given assignments and all of these assignments were correct. Linkage studies generally exclude such SNPs from analysis [10]. Finally LODE procedure can be used for checking the integrity of assembly by sampling and reassigning the positions of SNPs as shown in the result section.

The LODE procedure described here is complementary to other commonly used methods to assemble maps, including linkage maps and physical maps such as Fluorescence in situ hybridization (FISH) and Radiation Hybrid mapping [13], but offers significant advantages over these methods since they are very laborious, may have limited resolution, and often require highly specialized resources [22-25]. The comparative advantages and limitations of using LODE mapping are discussed in detail below.

The building of linkage maps for genome assemblies has the advantage that *de novo *ordering of markers can result in robust framework maps, but such maps required information from often large and specific resource populations. Indeed linkage maps have been assembled for many species including a broad range of markers (cattle [8], pig [26], sheep [27], mouse [28], chicken [29] and human [30]). In the case of mouse [31] was able to place SNP markers at a resolution and accuracy of 0.3 Mb by linkage mapping. Most resource populations do not have sufficient power to treat each marker in a high density map as a framework reference point (anchor marker) as described by Ott *et al*. [32] in their guidelines for developing linkage maps. Recently Arias *et al*. [10] reported on the construction of a bovine hybrid linkage-map by combining linkage and physical map (Btau4.0) information. However, of the 9,713 SNPs genotyped, 2,946 (30.3%) could not be assigned to the linkage map for quality control reasons. Furthermore 743 (9.4%) of the 7,822 markers assembled for mapping could not be positioned. In contrast the LODE procedure was able to place 4,688 out of 5,314 SNPs in a data set of 73,569 SNPs which is the largest panel of bovine SNPs which can currently be assembled from commercially available SNP arrays.

Integrated maps and comparative maps are frequently used to build interim maps for the species in the absence of a completed genome assembly [33,14,34]. BLAST procedure is commonly used to align sequence and when combined with LiftOver can make inference about marker position and order. However, this procedure is highly inefficient when compared to direct mapping such as LODE. For example, out of 4,688 SNPs successfully mapped by LODE, only 230 would have been mapped successfully using BLAST and LiftOver from human assembly to bovine assembly. Lewin *et al*. [7] highlighted the limitations and conundrum of using comparative mapping information for building maps and emphasised the importance of developing independent species specific maps for discovery of conserved chromosome segments and evolutionary breakpoint regions.

Despite the array of tools available for constructing genetic and physical maps, a large number of SNPs and scaffolds remain unpositioned which is likely to be common for most species in which genome assembly is being undertaken (chicken [35], dog [36], cat [37], pig [38] and many other species [1]). As such, the LODE mapping procedure offers a significant additional tool for completing genome maps and assemblies. LODE procedure relies on the linkage disequilibrium information from the unrelated samples from the population and does not require a specific resource population. A reliable estimate of *r*^2 ^can be obtained from a minimum sample size of 75 unrelated individuals [16] which can be found in many diversity and association studies.

However, despite the high degree of accuracy of placement, the LODE procedure still only provides an approximation of the exact localisation (precision) of SNPs within a chromosome, since it is dependent on the accuracy of prior genome assembly as a reference framework and the density of known SNPs to allow positioning of unknown SNPs. Hence, the precision of positioning SNPs with the LODE procedure will increase with increasing SNP density and accuracy of the sequence map. However, quality of the assembly can be assessed by using the LODE procedure to confirm the location of SNP markers with assigned positions, and provides for an independent cross check as shown in the result section. The initial density of marker maps, in order for the LODE procedure to be effective, will depend upon the extent of LD in the population which is often population specific. In the case where no reference positions are available as in the case of *denovo *genome sequencing and mapping, using *D' *as a measure of LD will be useful for LODE mapping (Solkner et al. in preparation). It is recommended to always test the LODE strategy on a panel of mapped SNPs with known positions, before applying the procedure to unmapped SNPs; and, if necessary, to alter some of the thresholds criteria.

LODE procedure can also be very helpful in refining sequence and genetic maps for species where comparative genome assemblies are used to build a virtual assembly for the species of interest, such as has recently been done for sheep [33]. Population wide (across or within breeds) LD information from high density SNP data (see the ISGC website http://www.sheephapmap.org/) can be used to place and validate SNP locations, and order of unplaced scaffolds where they contain SNPs with appropriate genotype information. The LODE procedure is likely to be of significance in the future as developments in next-generation sequencing technologies are providing deep sequencing coverage at an affordable price [39-41]. These platforms generally provide enormous information on new SNPs from short sequence reads [4,6] but these short sequence reads, at present, can only be assembled into short scaffolds. Genotyping SNPs with the advent of ultra-high genotyping platforms [42] will allow for LODE to integrate these short sequence scaffolds into the existing map information.

## Conclusion

The LODE procedure described in this study is an efficient and accurate procedure for positioning SNPs, and offers a means for positioning of unordered scaffolds containing SNPs. The LODE procedure will be helpful in refining genome sequence and checking assemblies, especially those being created from next-generation sequencing where high-throughput SNP discovery and genotyping populations are components of genome analysis.

## Authors' contributions

MSK conceived the method and the study, contributed in its design, data collection, analysis and was the primary author for assembling the manuscript. MH provided bioinformatics support in SNP positioning and comparative mapping. MN and JS participated in the method development and manuscript preparation. FWN contributed in the method development, interpretation and manuscript preparation. HWR is project leader and contributed in project concept, design, interpretation and manuscript preparation. All the authors have read and approved the final manuscript.

## Supplementary Material

Additional file 1 **The comparison of different threshold combinations for chromosomal assignments**. This file presents the results from alternate criteria that were tested during the development of the preferred strategy for the LODE procedure. Click here for file

Additional file 2 **Chromosome wise comparison of estimated (LODE) and known positions (Btau4.0) of 869 SNPs allocated a chromosomal position by LODE out a test set of 900 SNPs (MAF>0.05)**. This file contains 30 scatter plots, one for each bovine autosomes (1-29) and X-chromosome. Click here for file

Additional file 3 **The comparison of estimated (LODE) and known positions (Btau4.0) of 91 SNPs allocated a chromosomal position by LODE out a test set of 300 SNPs (0.01<MAF ≤ 0.05)**. The comparisons for positions are presented in a signal scatter plot for all the 91 SNPs combined over all chromosomes. Click here for file

Additional file 4 **List of 5,314 SNPs (MAF>0.01) from three SNPs chips which were unpositioned on bovine assembly Btau4.0**. This table presents the chromosomal positions for 4,688 of 5,314 SNPs estimated by LODE. The BLAST results against another bovine assembly UMD3.0 are also given. Click here for file

Additional file 5 **Assignment of 'Un' scaffolds to chromosomes by aligning all SNPs within 'Un' a scaffold to one chromosome by LODE procedure**. This file contains list of 494 "Un" scaffolds, chromsomal assignments by LODE, number of SNPs in each scaffold and the length of each scaffold. Click here for file

Additional file 6 **Detailed chromosome-wise comparison of chromosomal assignments of 52,987 SNPs repositioned by LODE procedure with original positions on Btau4.0**. This file contains 30 scatter plots, one for each bovine autosomes (1-29) and X-chromosome. Click here for file
