# Supplementary material for: Assignment of chromosomal locations for unassigned SNPs/scaffolds based on pair-wise linkage disequilibrium estimates
Source: BMC Bioinformatics. 2010 Apr 7;11:171. doi: 10.1186/1471-2105-11-171 (PMC2859757; doi:10.1186/1471-2105-11-171)
Supplement: Additional file 1 — The comparison of different threshold combinations for chromosomal assignments. This file presents the results from alternate criteria that were tested during the development of the preferred strategy for the LODE procedure. [file 1471-2105-11-171-S1.DOC]

Table. Comparison of different threshold combinations for chromosomal assignments.

**Test Set 1.**  SNPs MAF>0.05 (Total 900 SNPs):

|  | Correct assignments | Incorrect assignments | Thresholds |
| --- | --- | --- | --- |
| 1 | 883 | 16 | only based on *r^2^_max_* |
| 2 | 872 | 1 | *r^2^_max_* + *n_0.1_* |
| **3** | **869** | **1** | ***r^2^_max_* + *n_0.1_* + *n_0.1_*>2** |
| 4 | 847 | 1 | *r^2^_max_* + *n_0.1_* + *n_0.1_* >2 + 2^nd^ chromosome |

**Test Set 2.** SNPs 0.05<MAF>0.01 (Total 300 SNPs):

|  | Correct assignments | | | Incorrect assignments | Thresholds | |
| --- | --- | --- | --- | --- | --- | --- |
| 1 | | 177 | 112 | | | only based on *r^2^_max_* |
| 2 | | 144 | 37 | | | *r^2^_max_* + *n_0.1_* |
| 3 | | 128 | 10 | | | *r^2^_max_* + *n_0.1_* + *n_0.1_*>2 |
| **4** | | **91** | **1** | | | ***r^2^_max_* + *n_0.1_* + *n_0.1_* >2 +** 2^nd^ chromosome |

**Test Set 3.** SNPs with 0.001<MAF <0.01  (Total 300 SNPs):

|  | Correct assignments | | | Incorrect assignments | Thresholds | |
| --- | --- | --- | --- | --- | --- | --- |
| 1 | | 21 | 127 | | | only based on *r^2^_max_* |
| 2 | | 10 | 57 | | | *r^2^_max_* + *n_0.1_* |
| 3 | | 2 | 10 | | | ***r^2^_max_* + *n_0.1_* + *n_0.1_*>2** |
| 5 | | 2 | 2 | | | *r^2^_max_* + *n_0.1_* + *n_0.1_* >2 + 2^nd^ chromosome |

Where ***r^2^_max_*** and *n_0.1_*  and 2^nd^ chromosome (comparison with second best chromosome) are thresholds described in material and methods.
