# Supplementary material for: Assignment of chromosomal locations for unassigned SNPs/scaffolds based on pair-wise linkage disequilibrium estimates
Source: BMC Bioinformatics. 2010 Apr 7;11:171. doi: 10.1186/1471-2105-11-171 (PMC2859757; doi:10.1186/1471-2105-11-171)
Supplement: Additional file 2 — Chromosome wise comparison of estimated (LODE) and known positions (Btau4.0) of 869 SNPs allocated a chromosomal position by LODE out a test set of 900 SNPs (MAF>0.05). This file contains 30 scatter plots, one for each bovine autosomes (1-29) and X-chromosome. [file 1471-2105-11-171-S2.PDF]

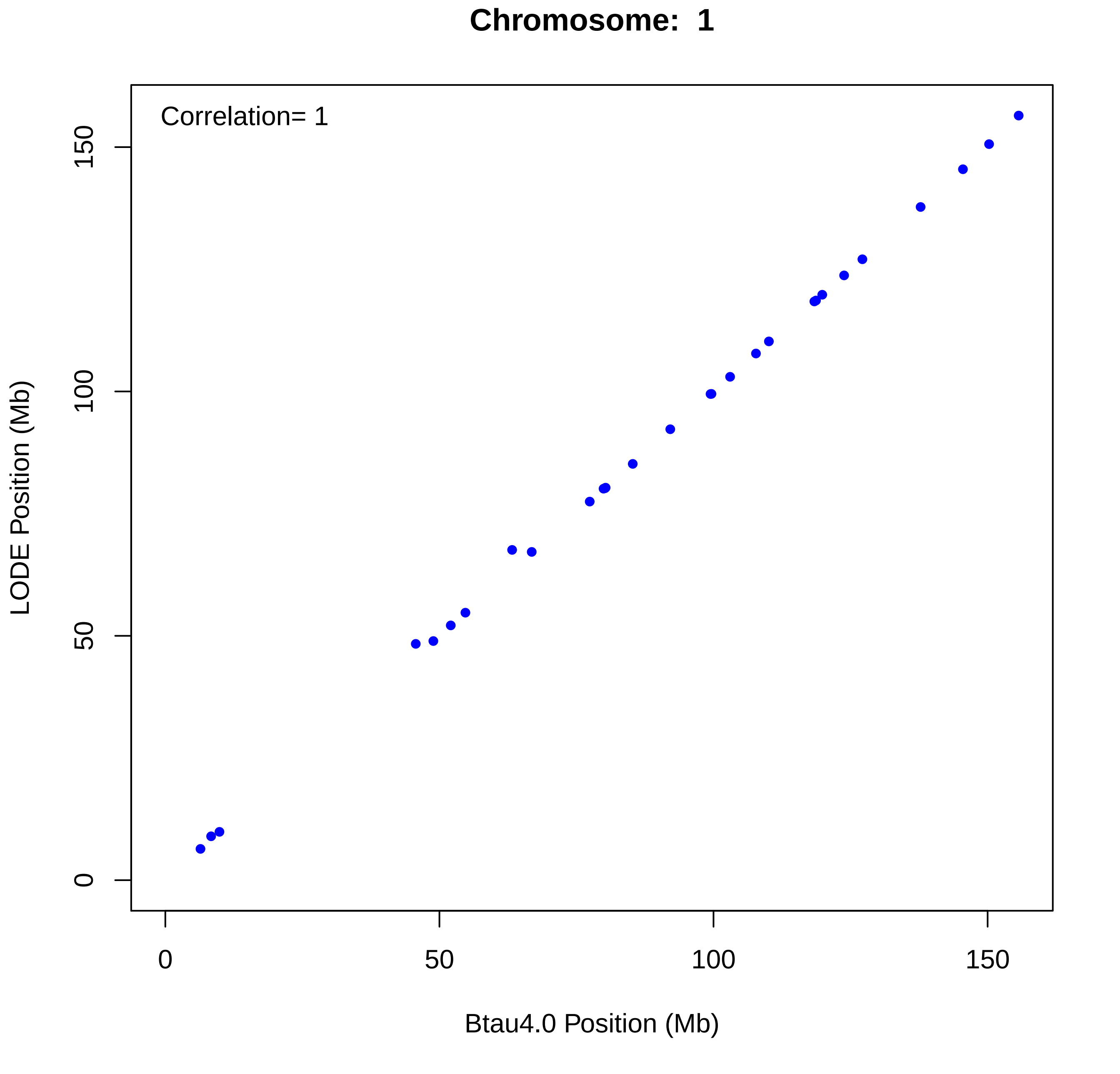

Chromosome: 1
Correlation= 1
LODE Position (Mb)
0
50
100
150
Btau4.0 Position (Mb)

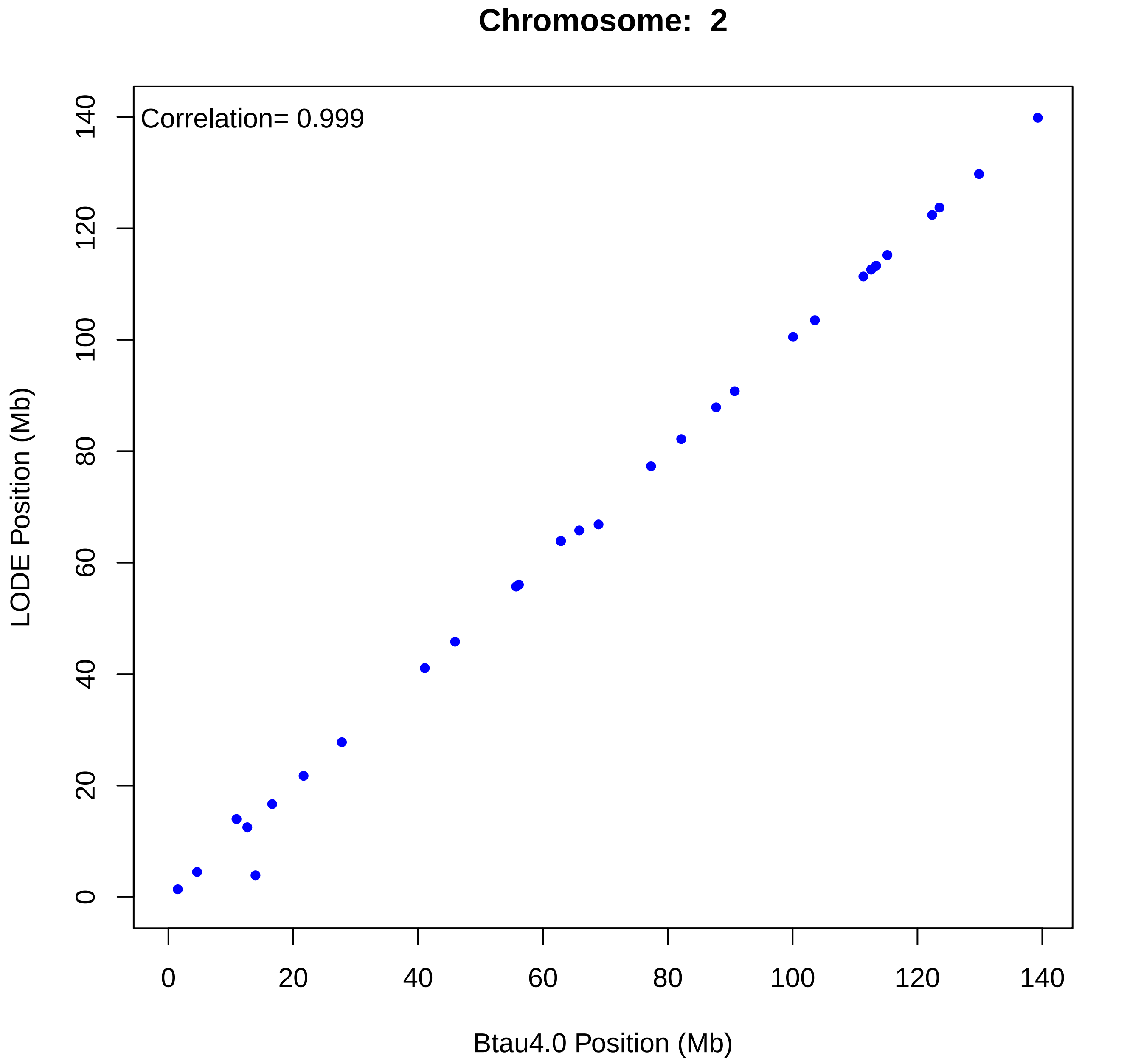

Chromosome: 2
Correlation= 0.999
LODE Position (Mb)
Btau4.0 Position (Mb)
0
20
40
60
80
100
120
140

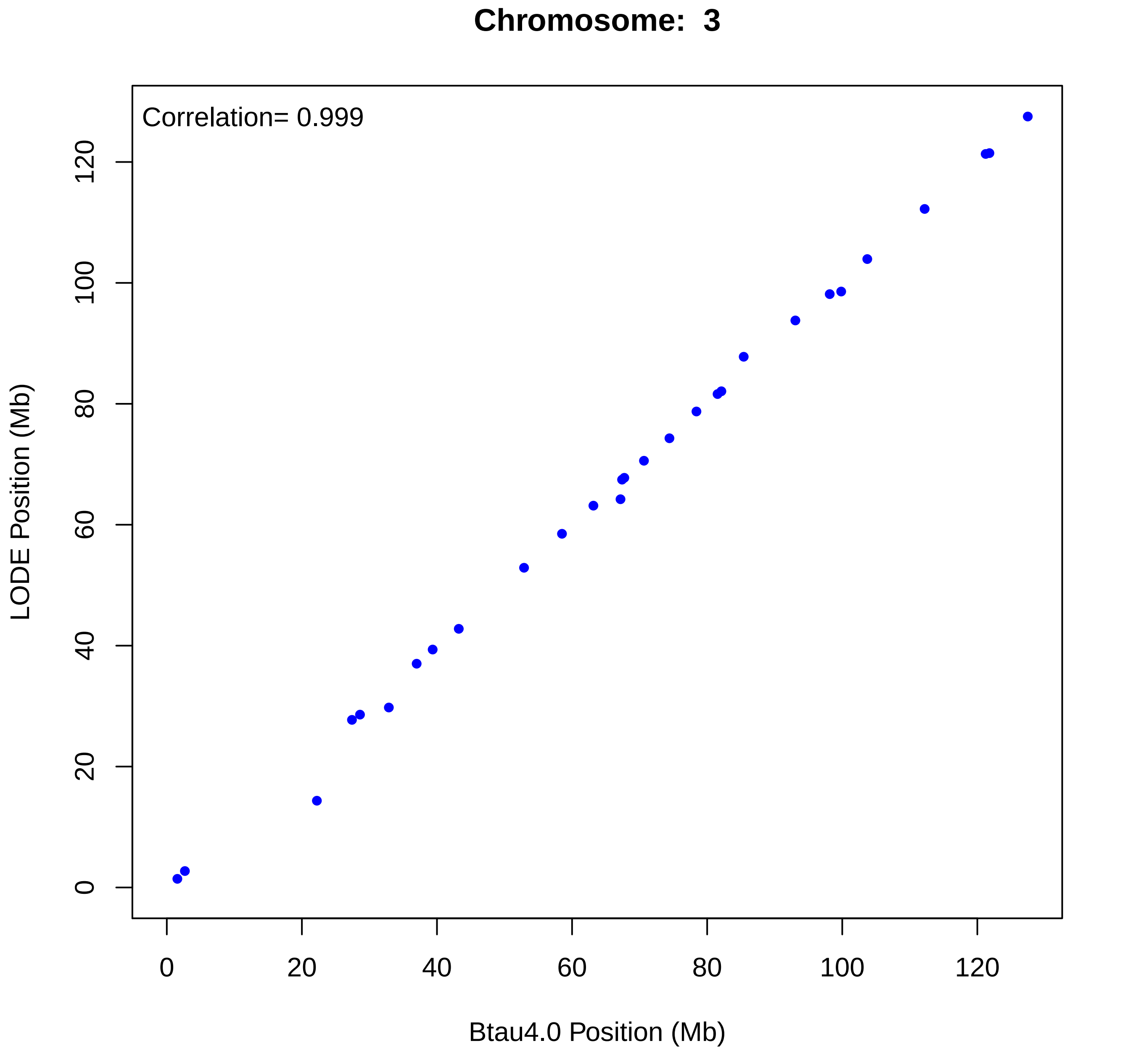

Chromosome: 3
Correlation= 0.999
LODE Position (Mb)
Btau4.0 Position (Mb)
0
20
40
60
80
100
120

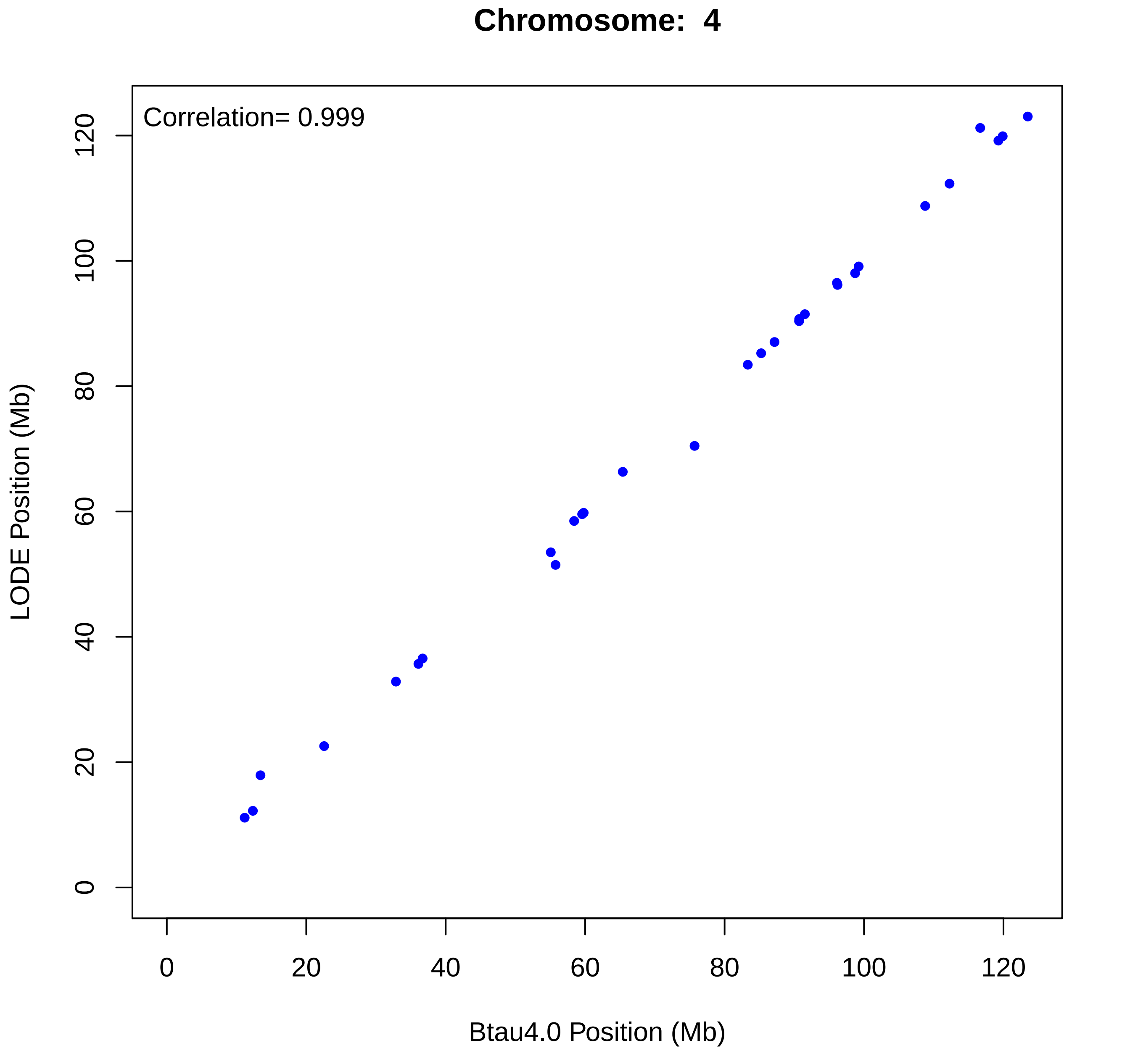

Chromosome: 4
Correlation= 0.999
LODE Position (Mb)
Btau4.0 Position (Mb)
0
20
40
60
80
100
120

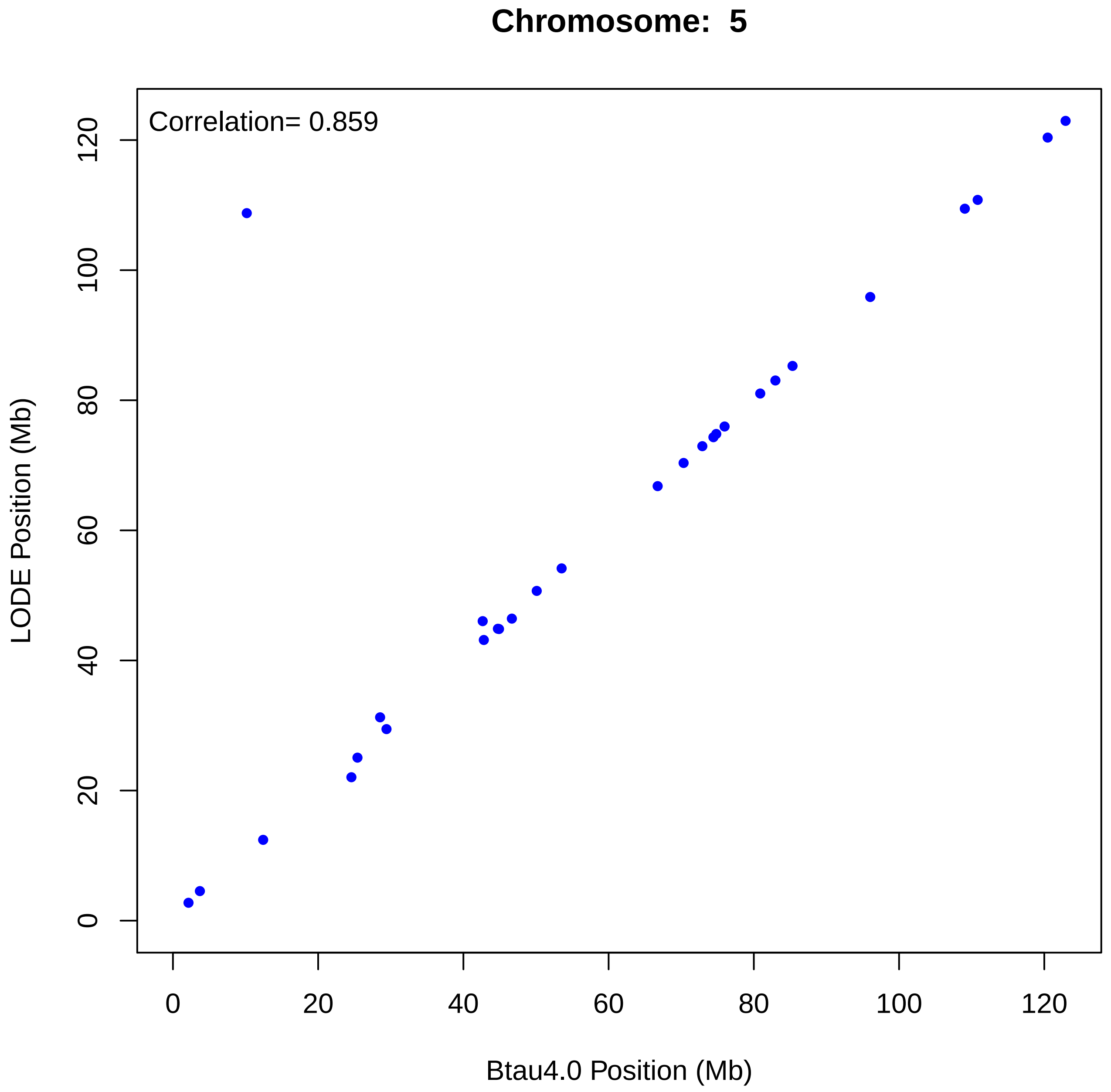

Chromosome: 5
Correlation= 0.859
LODE Position (Mb)
0
20
40
60
80
100
120
0
20
40
60
80
100
120
Btau4.0 Position (Mb)

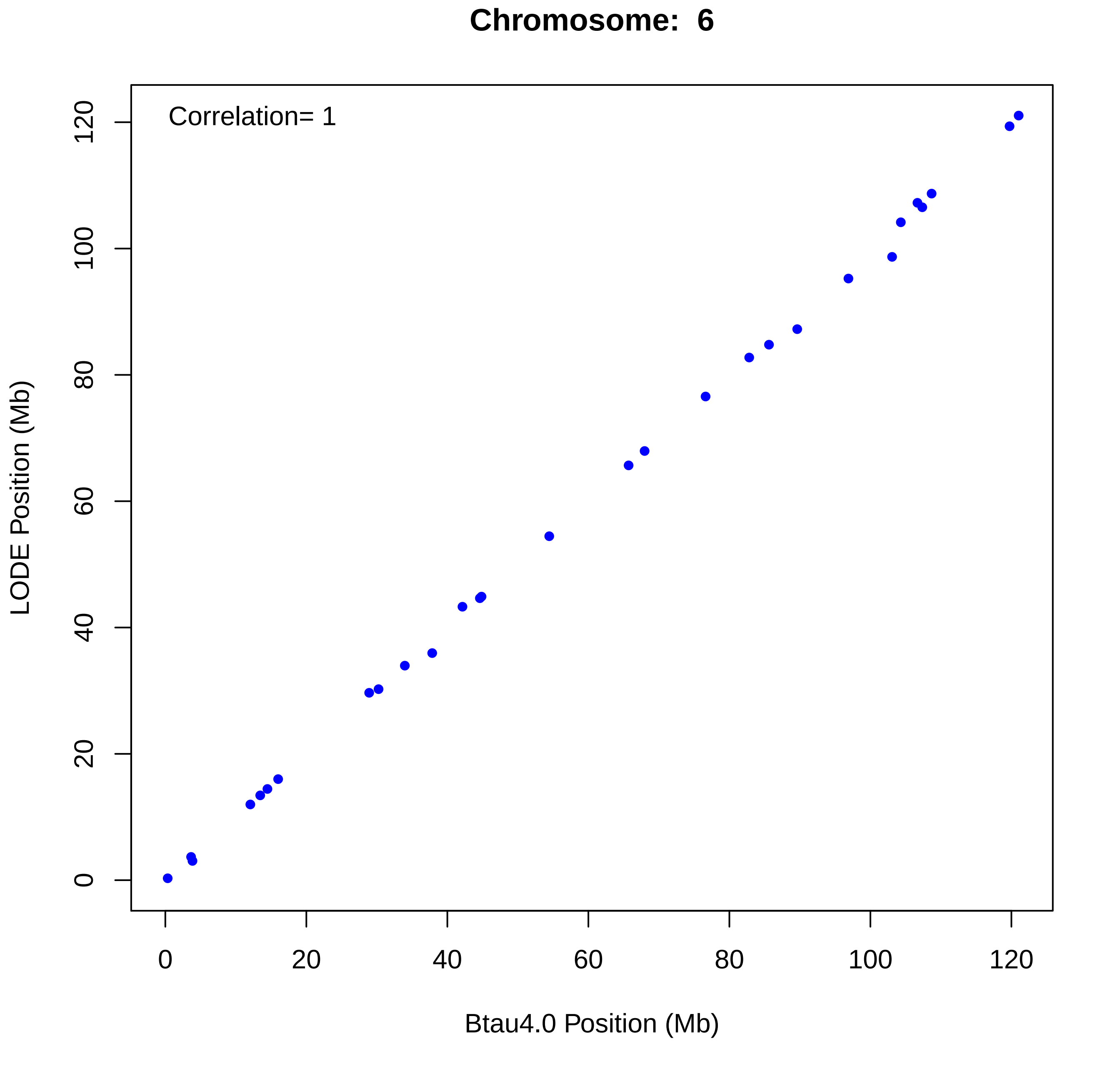

Chromosome: 6
Correlation= 1
LODE Position (Mb)
0
20
40
60
80
100
120
Btau4.0 Position (Mb)
0
20
40
60
80
100
120

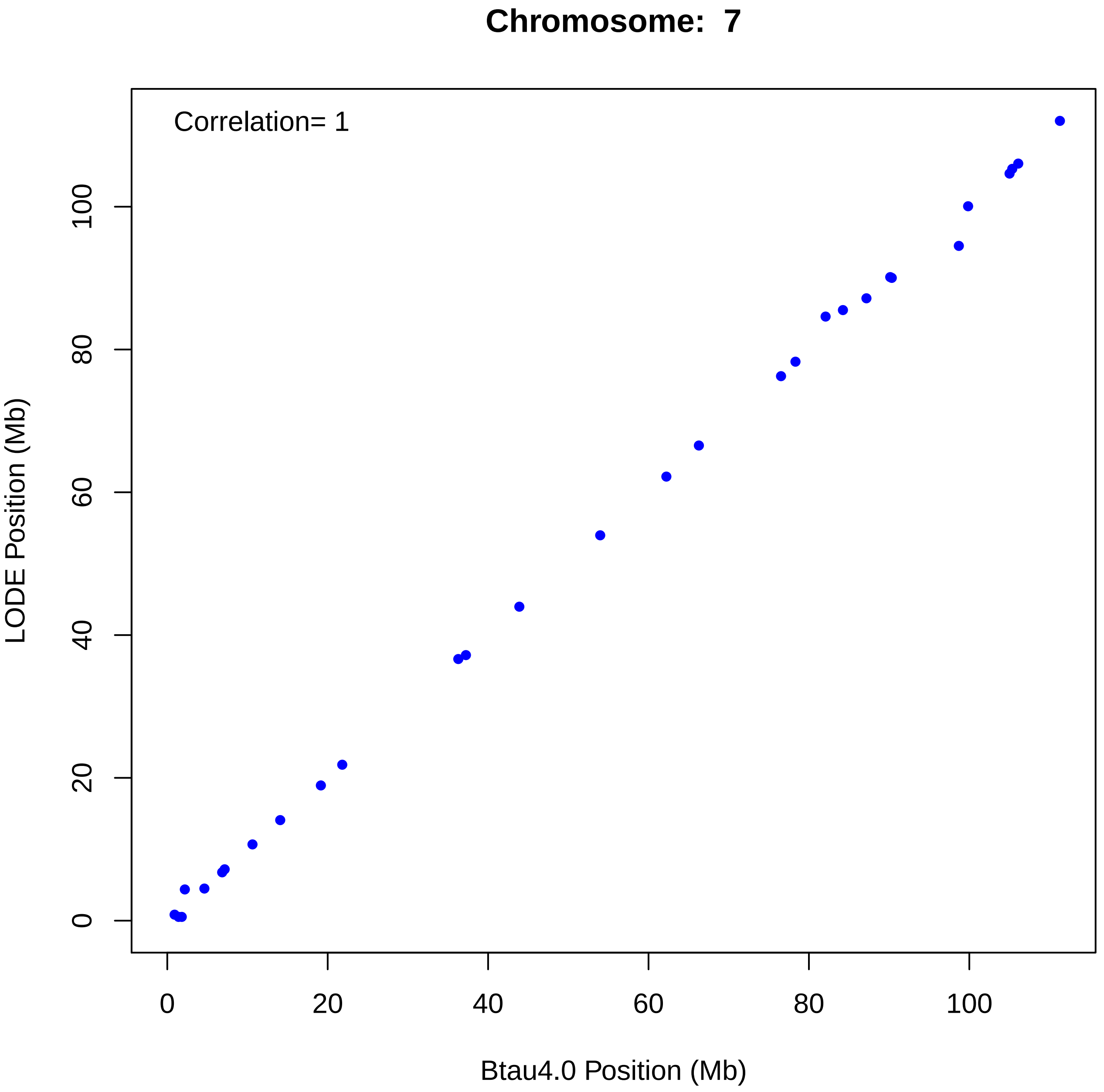
Chromosome: 7
Correlation= 1
LODE Position (Mb)
0
20
40
60
80
100
Btau4.0 Position (Mb)
0
20
40
60
80
100

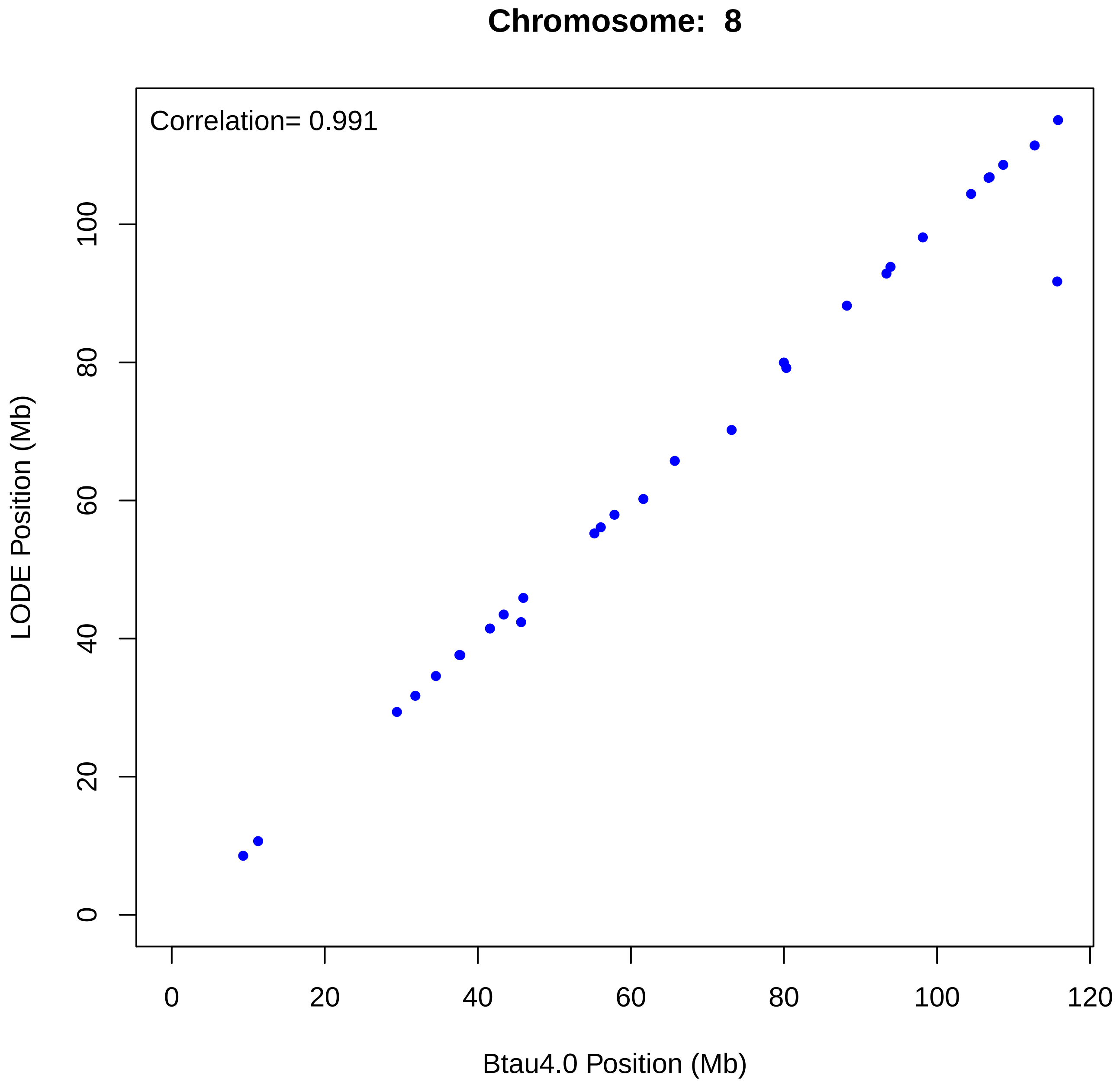

Chromosome: 8
Correlation= 0.991
LODE Position (Mb)
Btau4.0 Position (Mb)
0
20
40
60
80
100
120

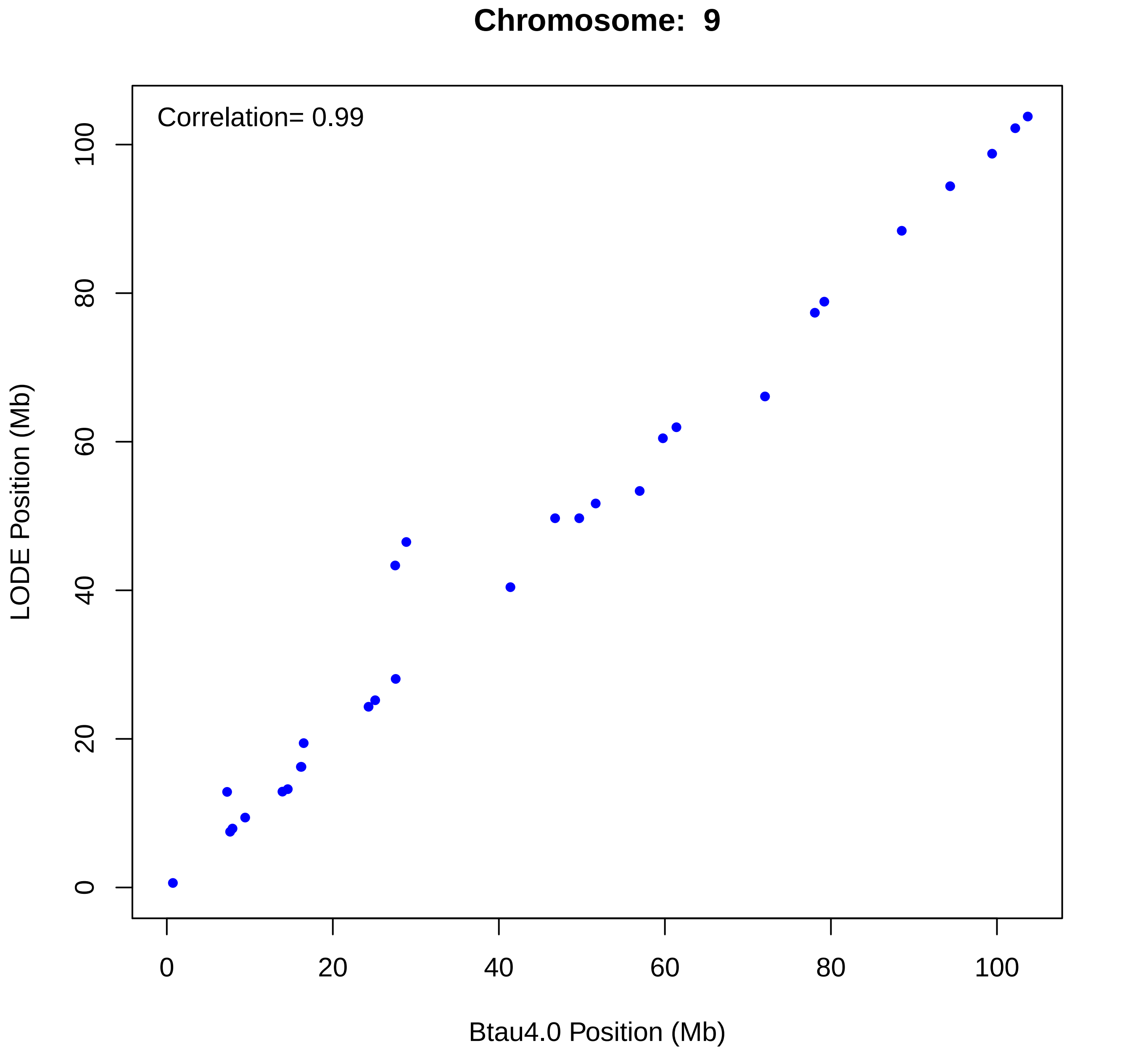

Chromosome: 9
Correlation= 0.99
LODE Position (Mb)
Btau4.0 Position (Mb)
0
20
40
60
80
100

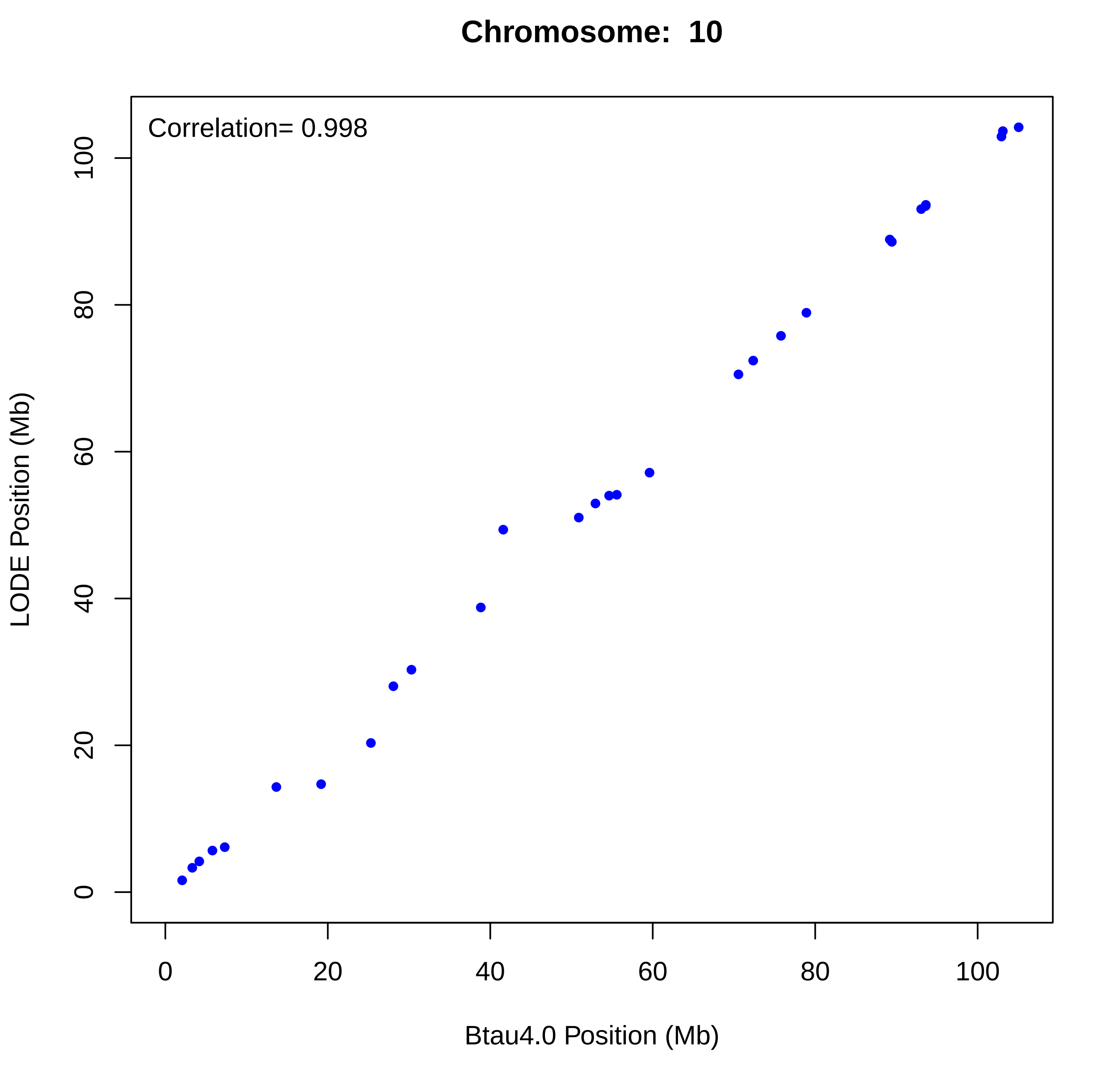
Chromosome: 10
Correlation= 0.998
LODE Position (Mb)
0
20
40
60
80
100
Btau4.0 Position (Mb)
0
20
40
60
80
100

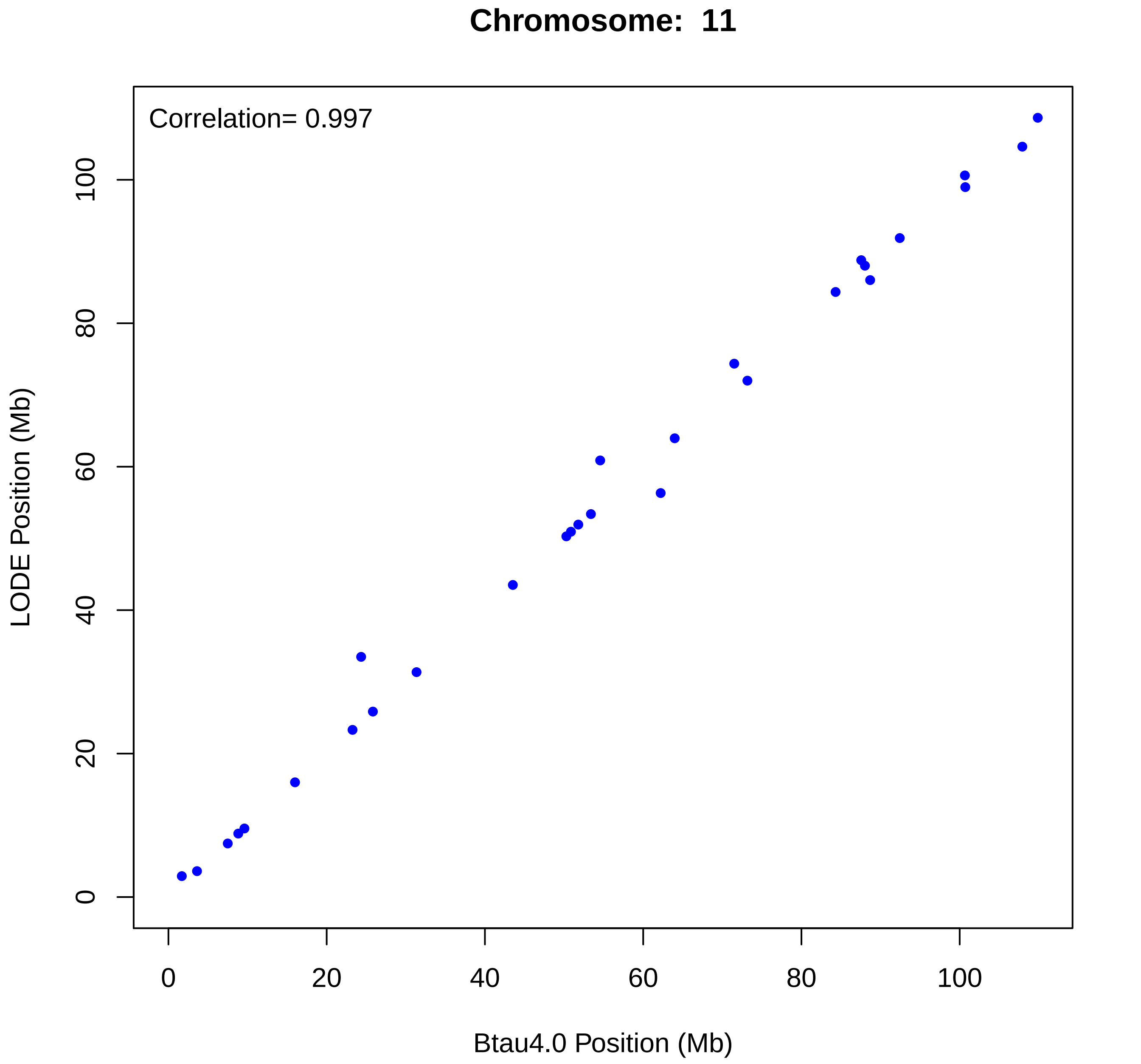

Chromosome: 11
Correlation= 0.997
LODE Position (Mb)
0
20
40
60
80
100
Btau4.0 Position (Mb)
0
20
40
60
80
100

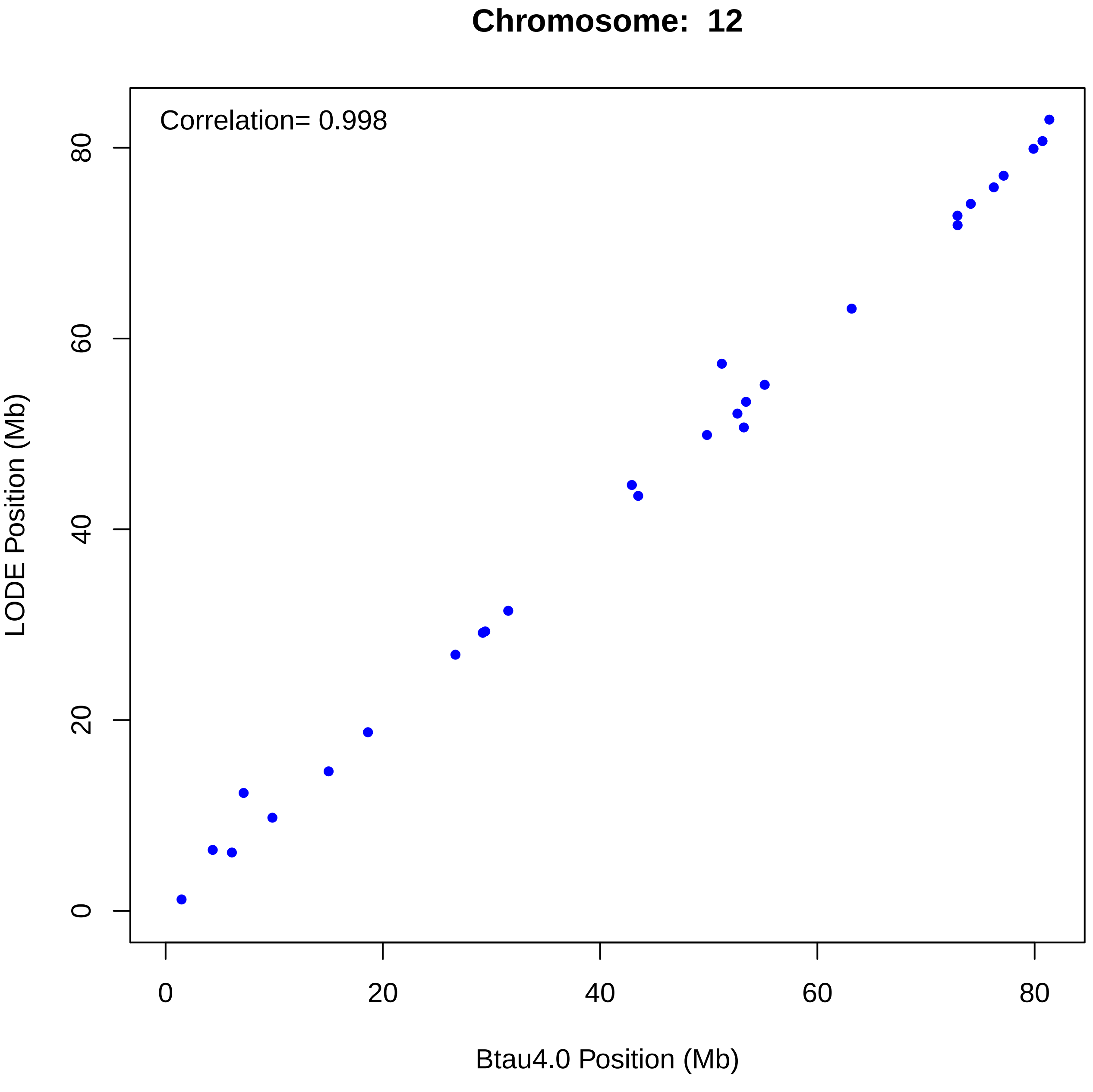

Chromosome: 12
Correlation= 0.998
LODE Position (Mb)
Btau4.0 Position (Mb)
0
20
40
60
80

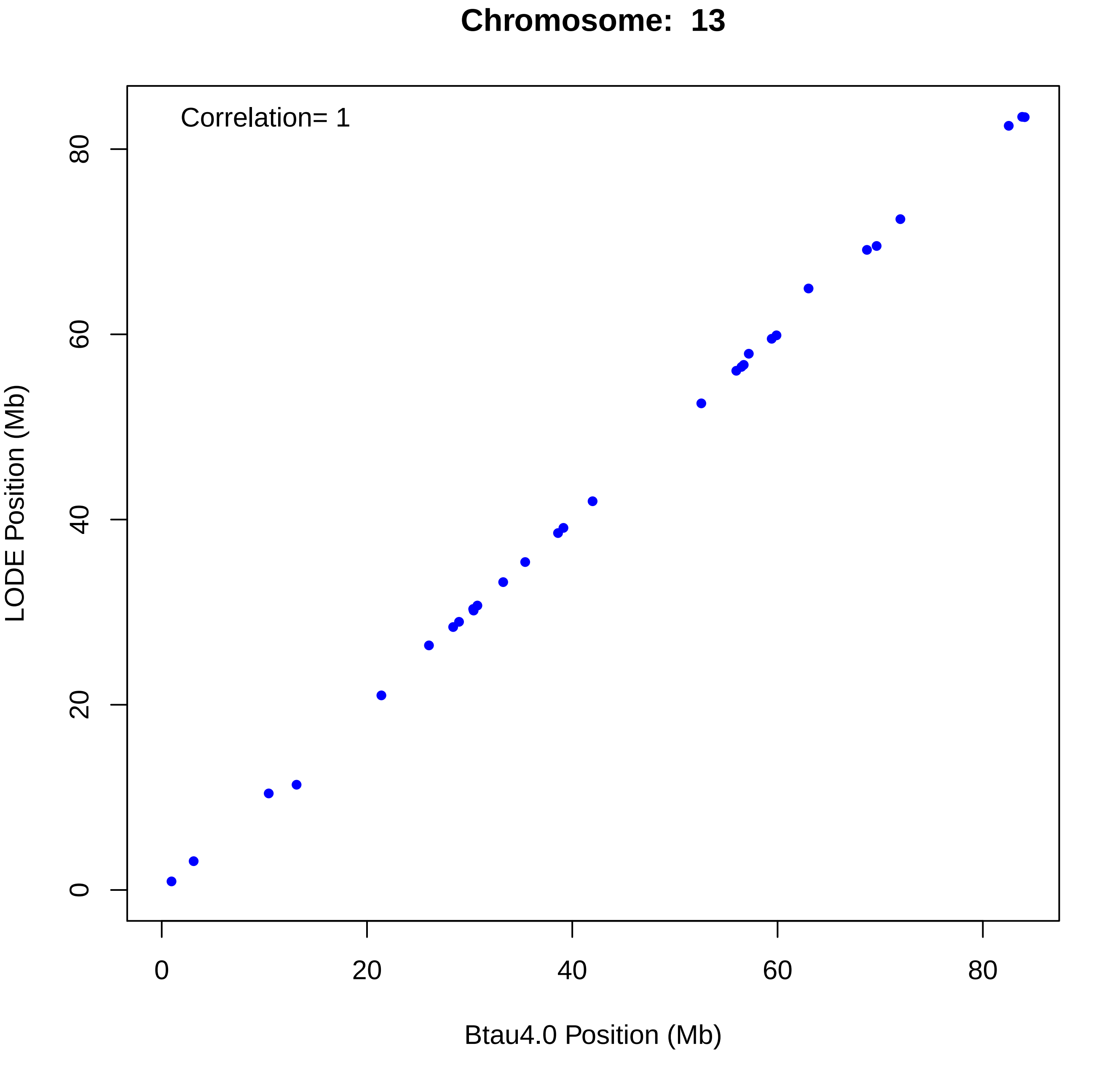

Chromosome: 13
Correlation= 1
LODE Position (Mb)
Btau4.0 Position (Mb)
0
20
40
60
80

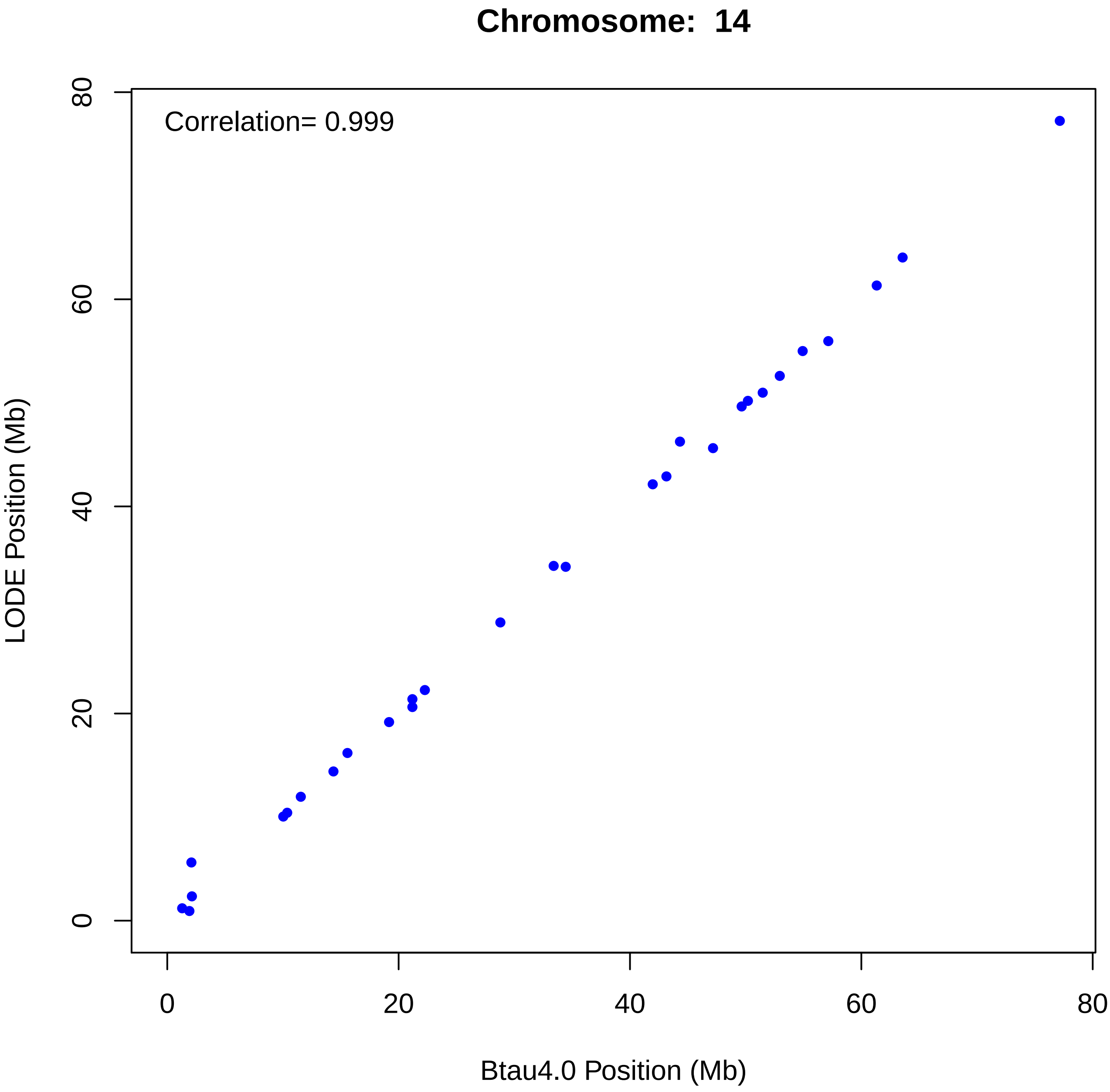
Chromosome: 14
Correlation= 0.999
LODE Position (Mb)
Btau4.0 Position (Mb)
0
20
40
60
80
0
20
40
60
80

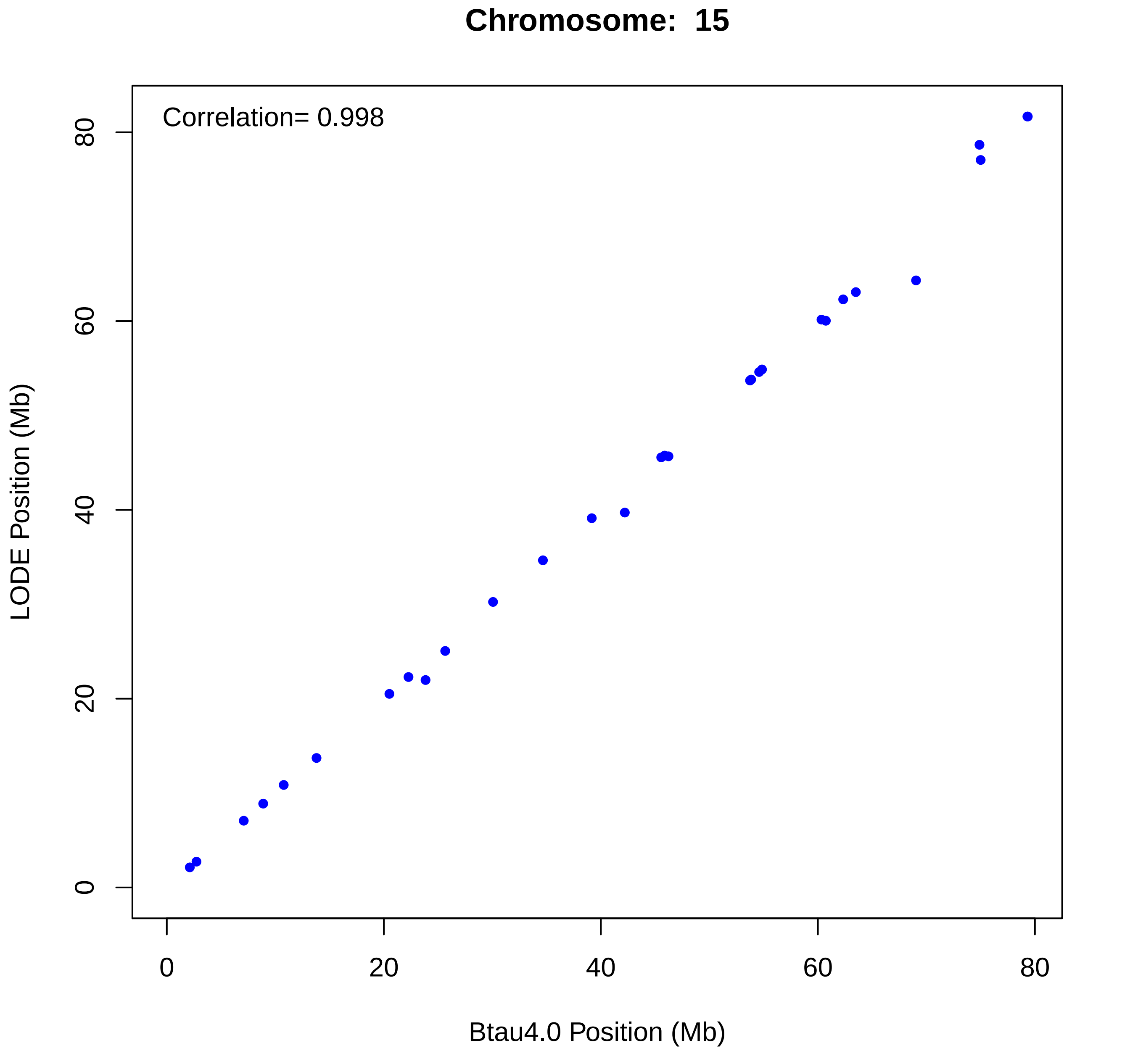

Chromosome: 15
Correlation= 0.998
LODE Position (Mb)
Btau4.0 Position (Mb)
0
20
40
60
80

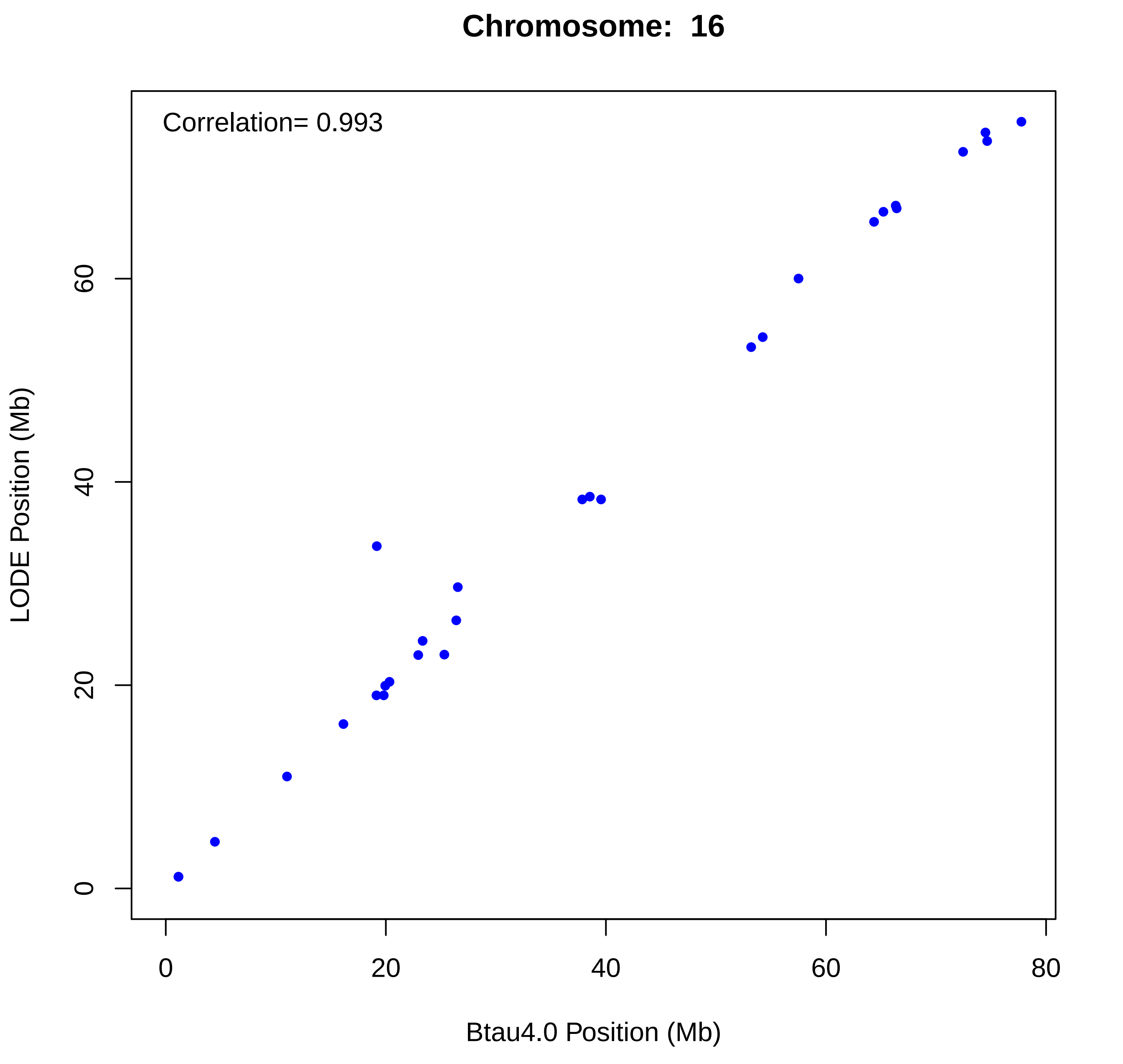
Chromosome: 16
Correlation= 0.993
LODE Position (Mb)
0
20
40
60
0
20
40
60
80
Btau4.0 Position (Mb)

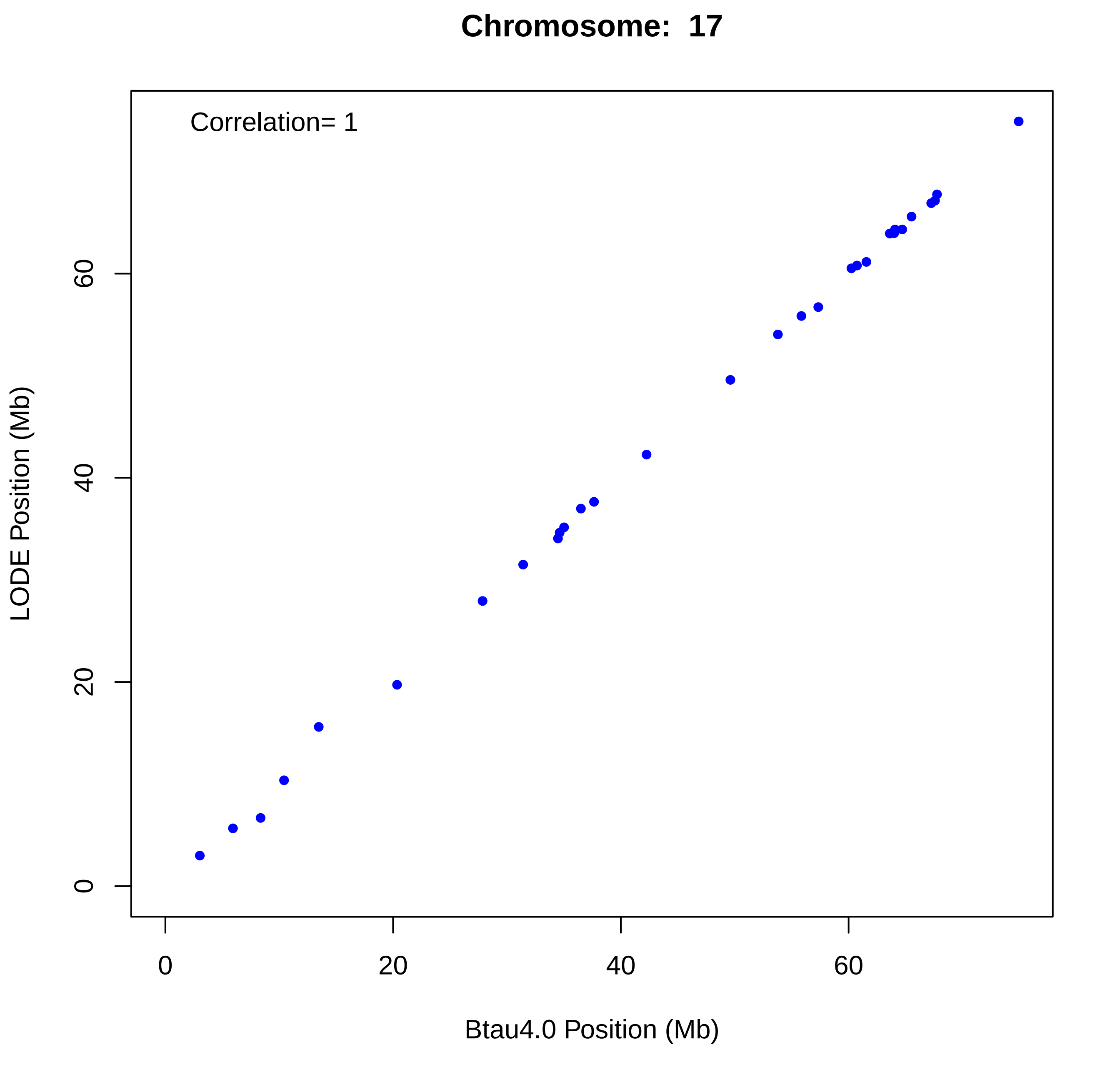
Chromosome: 17
Correlation= 1
LODE Position (Mb)
Btau4.0 Position (Mb)
0
20
40
60
0
20
40
60

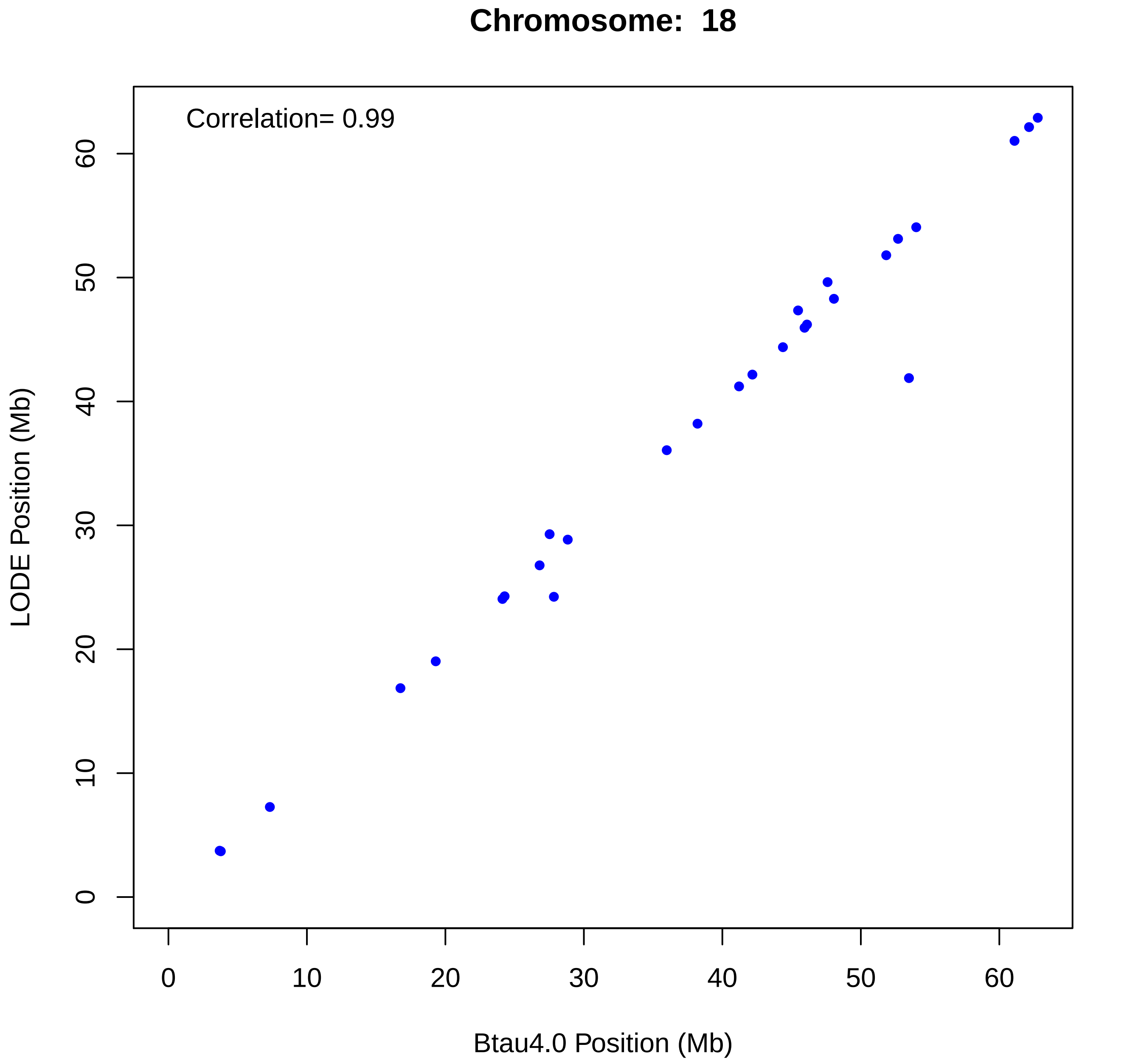

Chromosome: 18
Correlation= 0.99
LODE Position (Mb)
Btau4.0 Position (Mb)
0
10
20
30
40
50
60

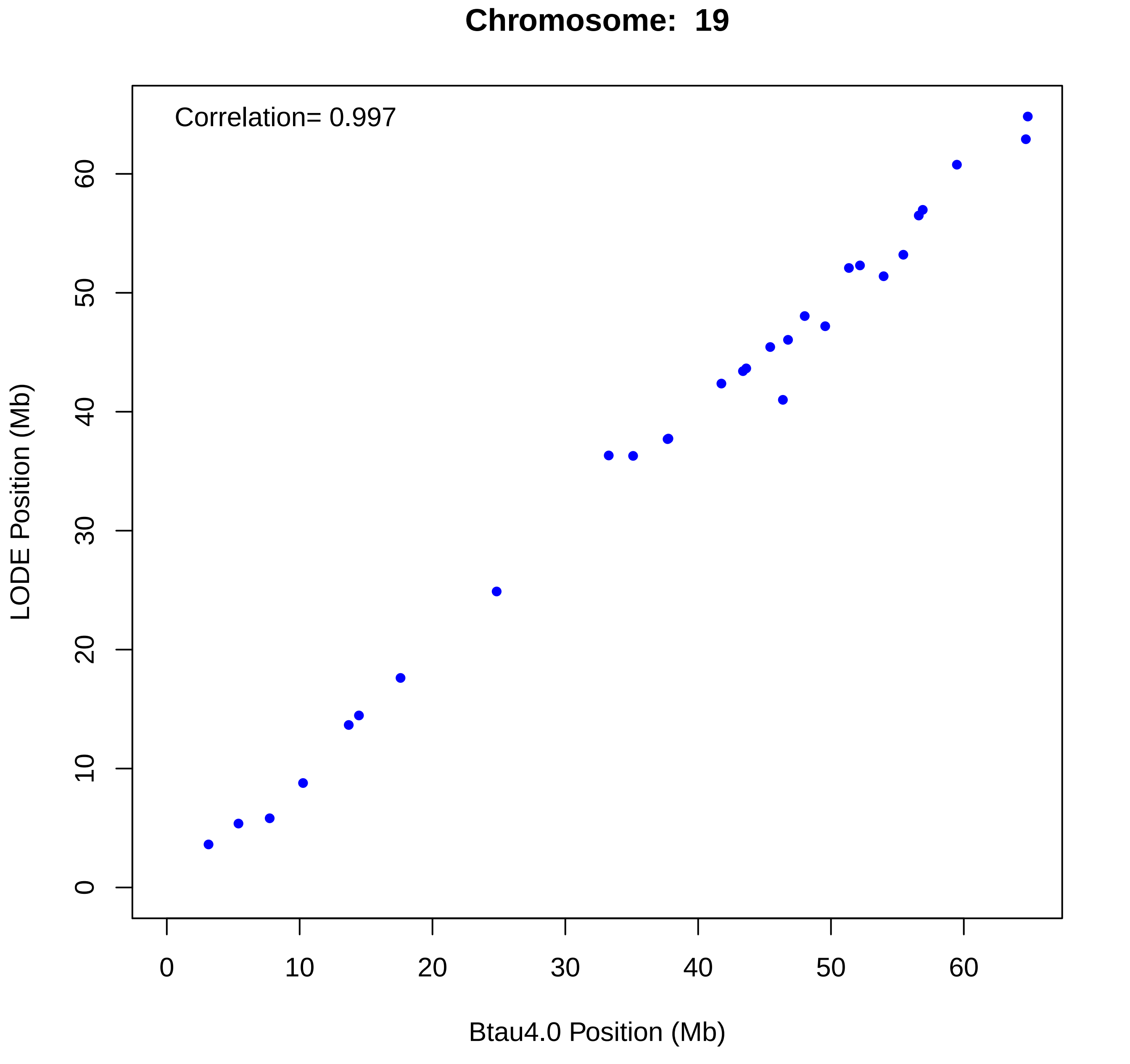
Chromosome: 19
Correlation= 0.997
LODE Position (Mb)
0
10
20
30
40
50
60
0
10
20
30
40
50
60
Btau4.0 Position (Mb)

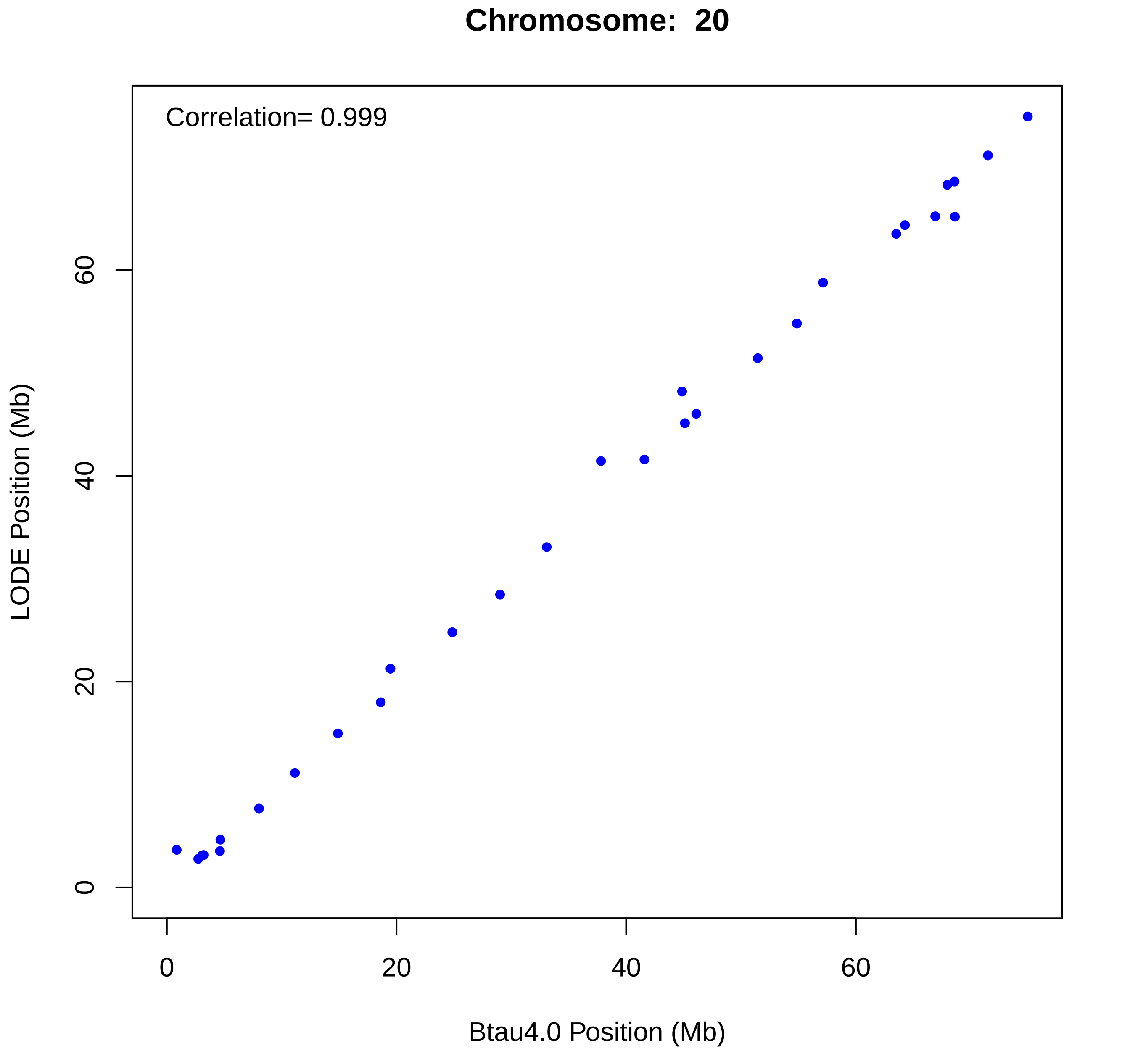

Chromosome: 20
Correlation= 0.999
LODE Position (Mb)
Btau4.0 Position (Mb)
0
20
40
60

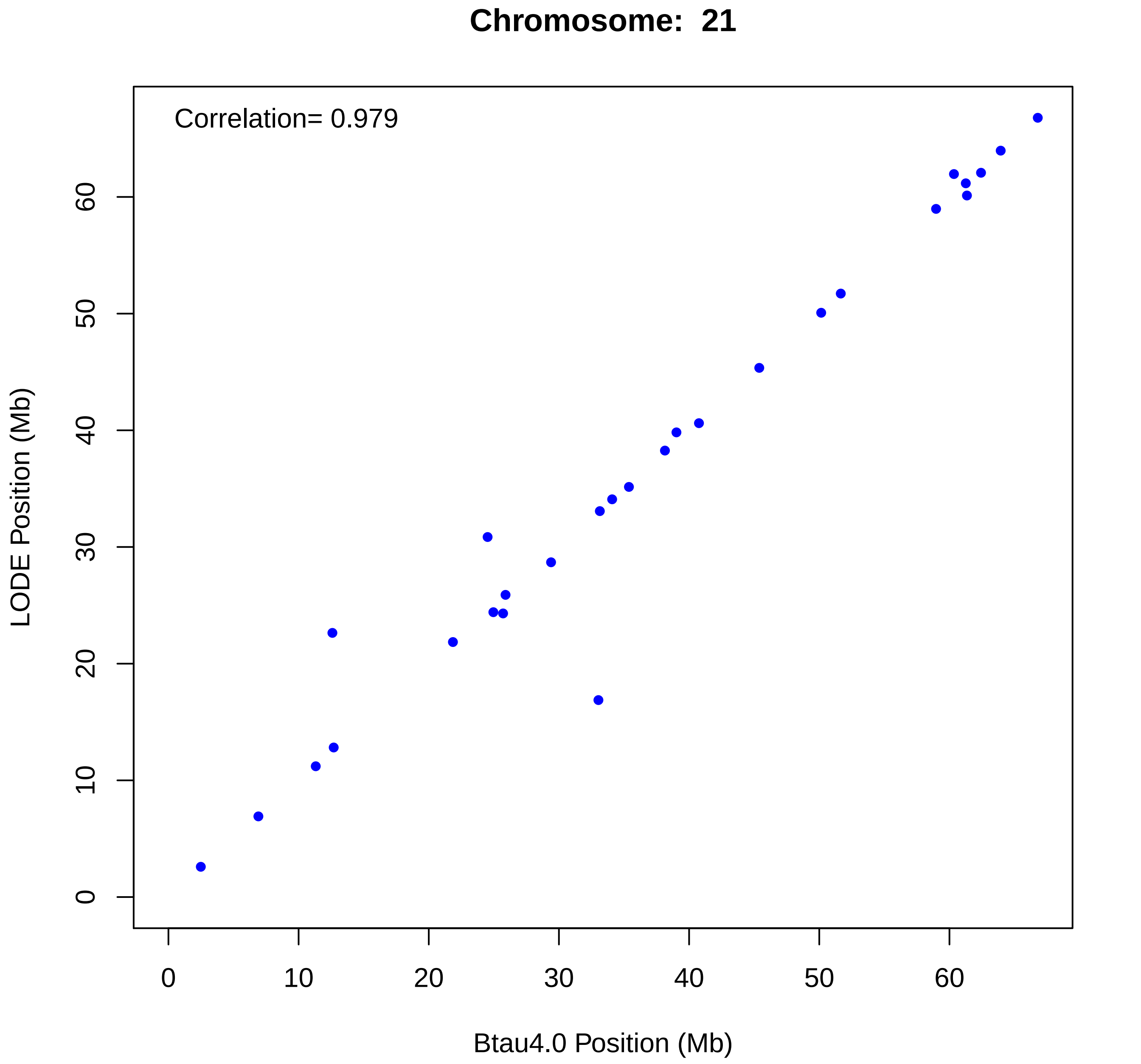
Chromosome: 21
Correlation= 0.979
LODE Position (Mb)
Btau4.0 Position (Mb)
0
10
20
30
40
50
60

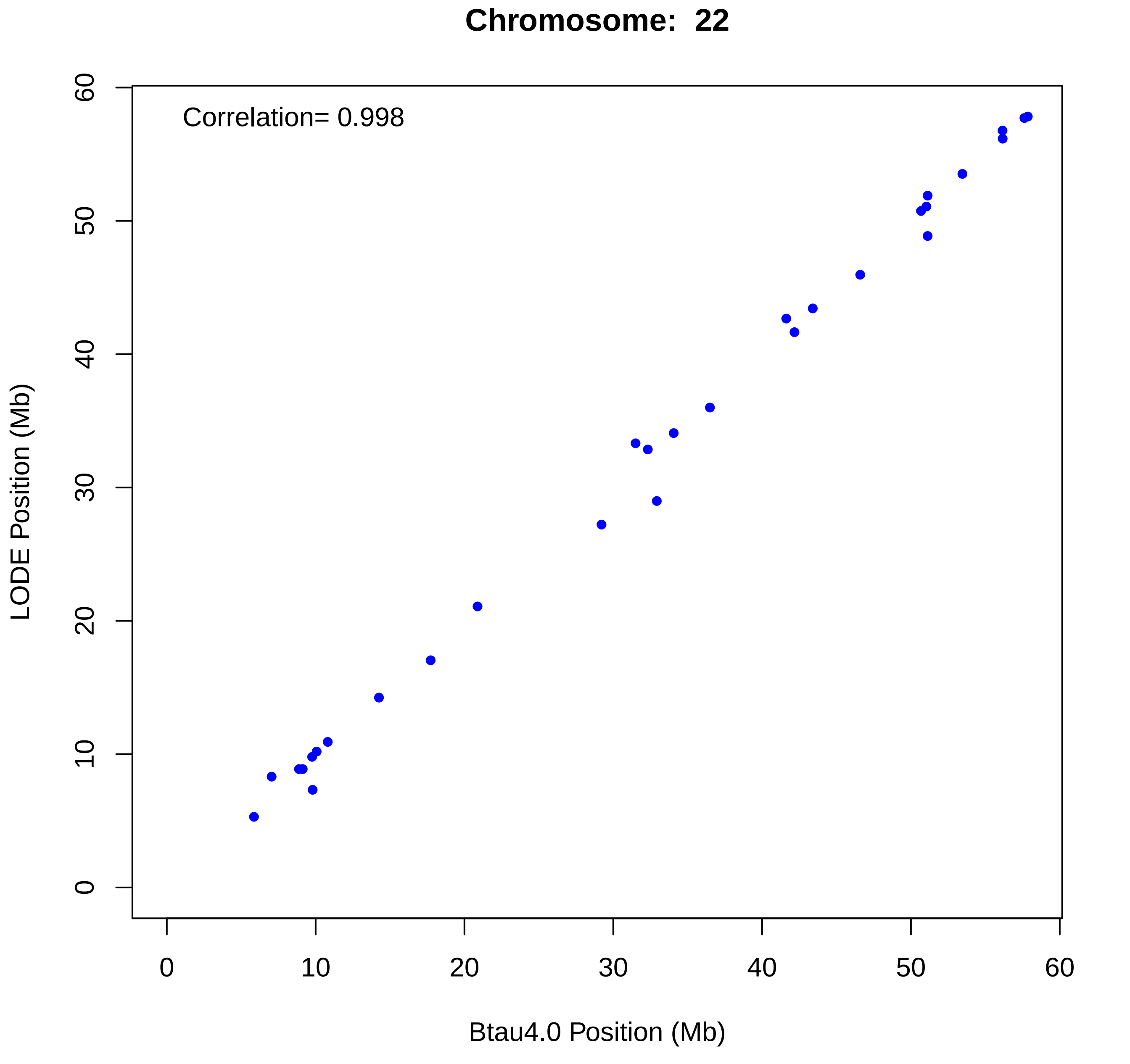
Chromosome: 22
Correlation= 0.998
LODE Position (Mb)
Btau4.0 Position (Mb)
0
10
20
30
40
50
60
0
10
20
30
40
50
60

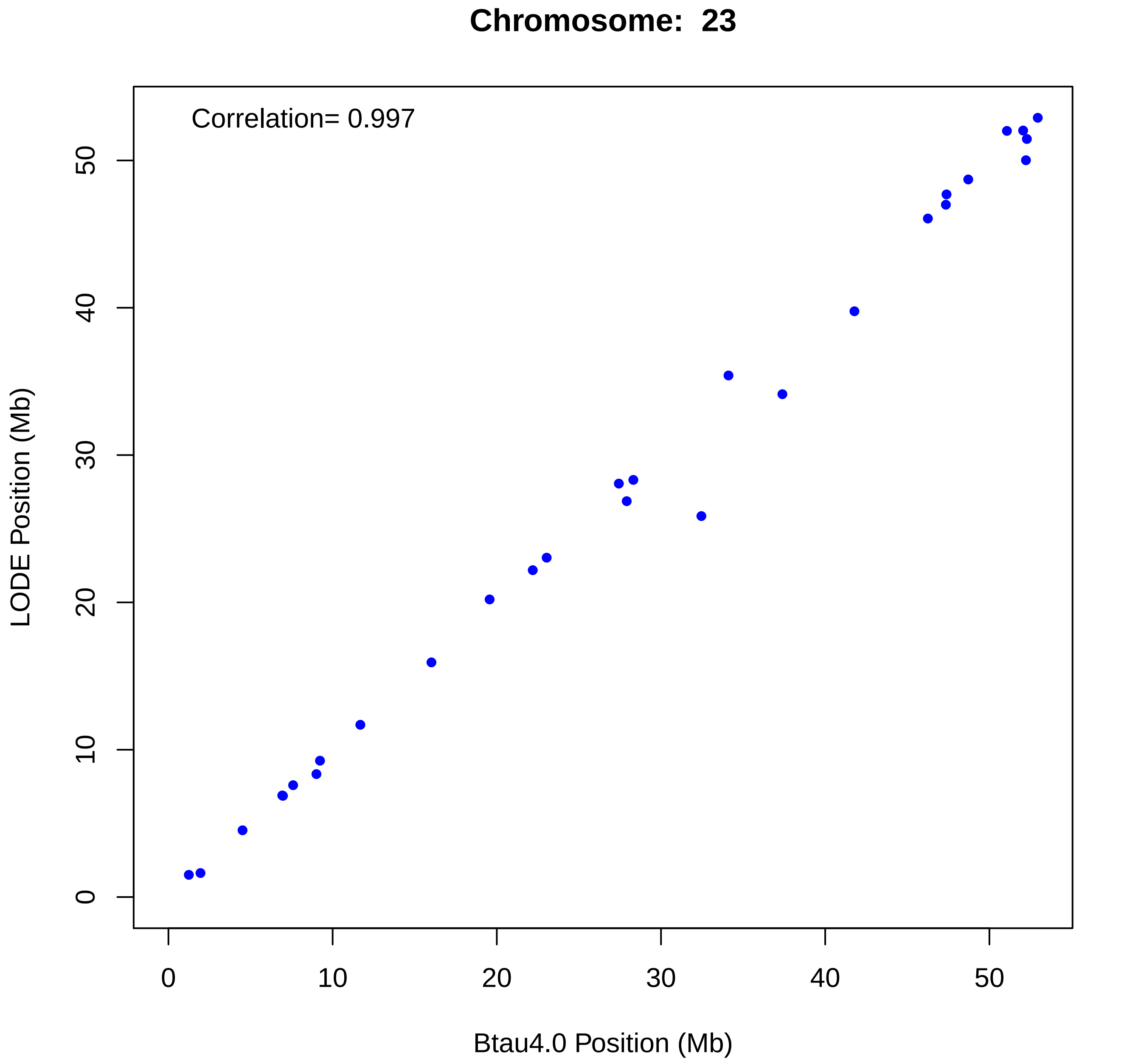
Chromosome: 23
Correlation= 0.997
LODE Position (Mb)
Btau4.0 Position (Mb)
0
10
20
30
40
50
0
10
20
30
40
50

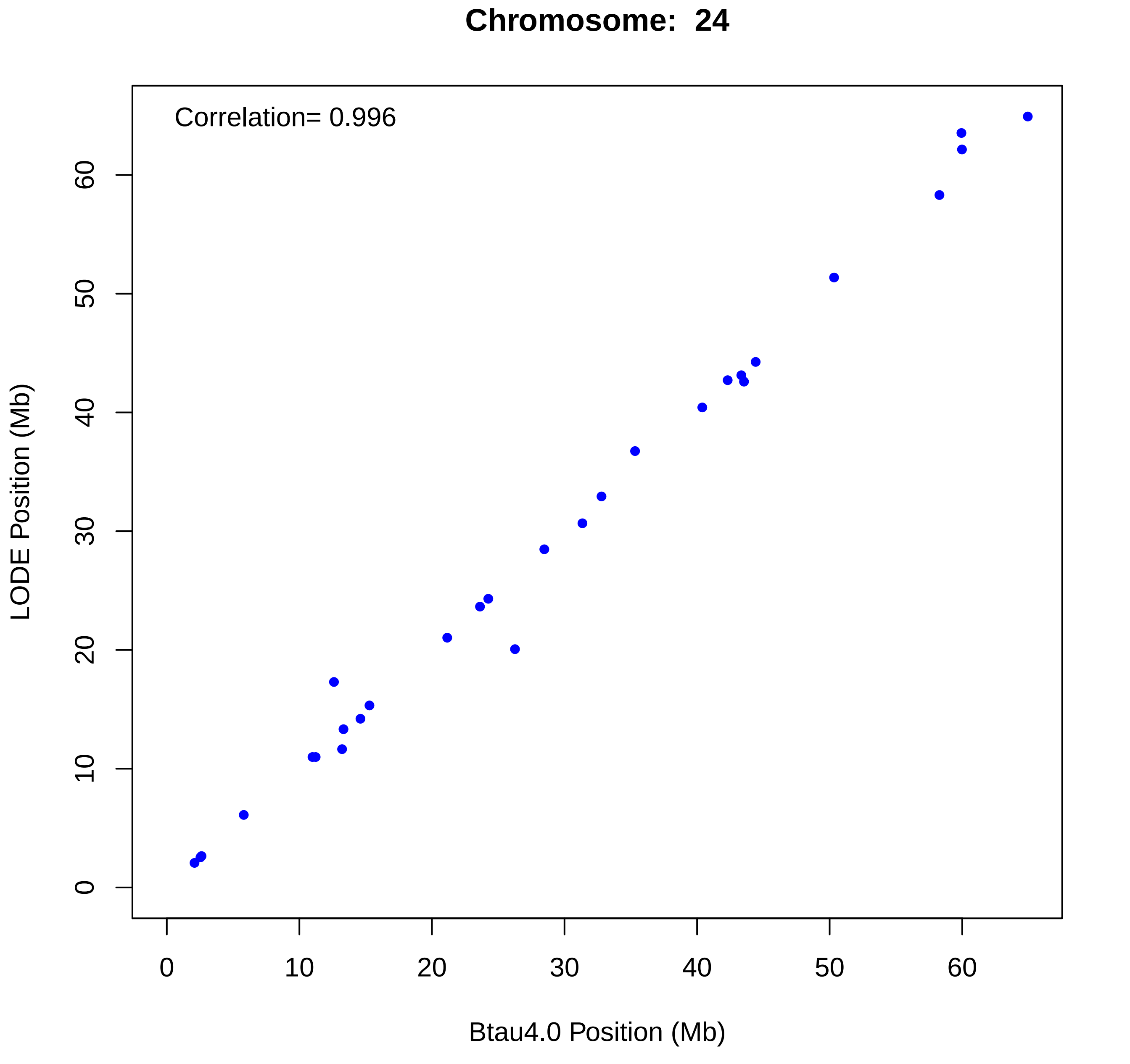

Chromosome: 24
Correlation= 0.996
LODE Position (Mb)
0
10
20
30
40
50
60
Btau4.0 Position (Mb)

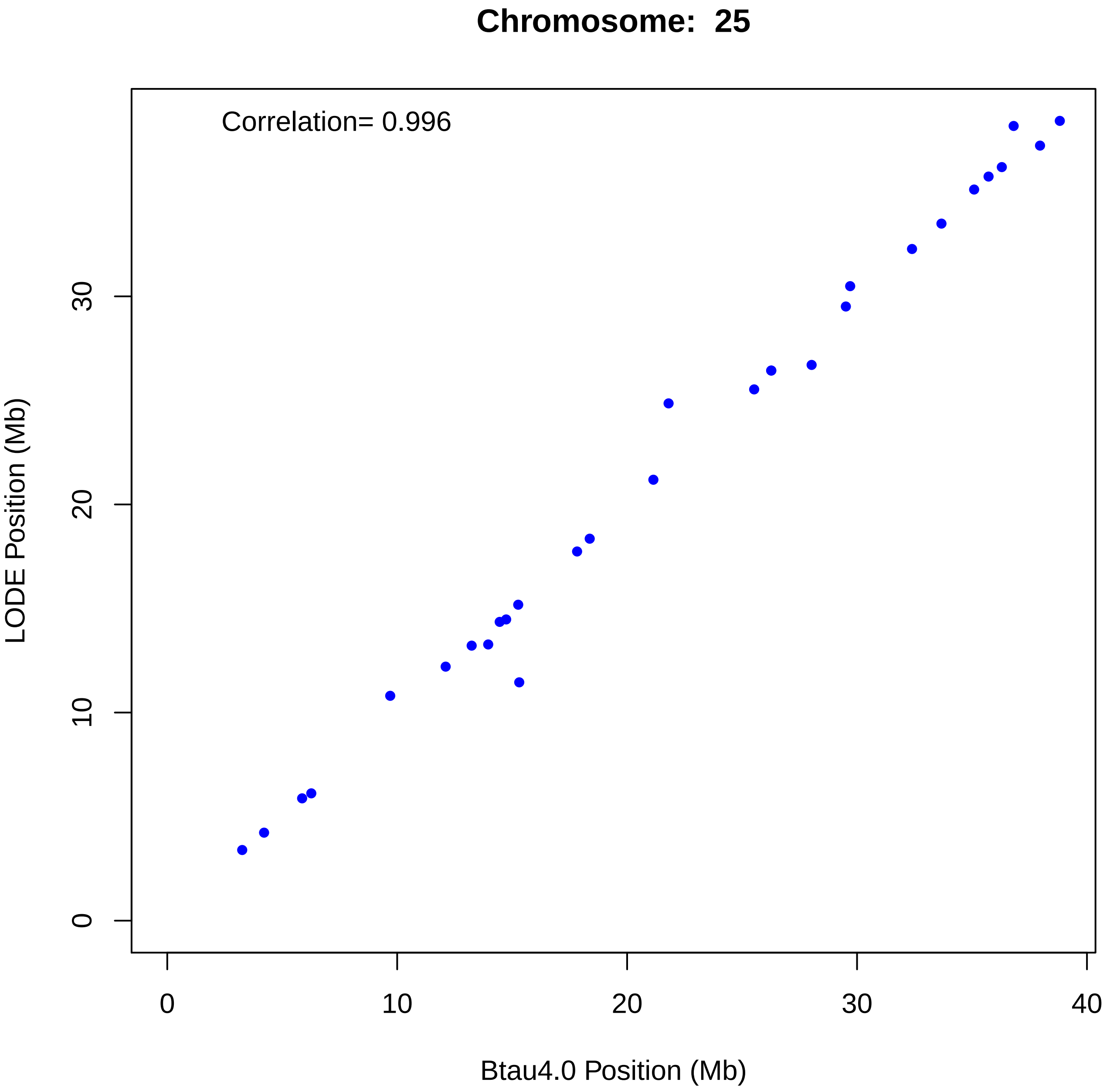

Chromosome: 25
Correlation= 0.996
LODE Position (Mb)
0
10
20
30
0
10
20
30
40
Btau4.0 Position (Mb)

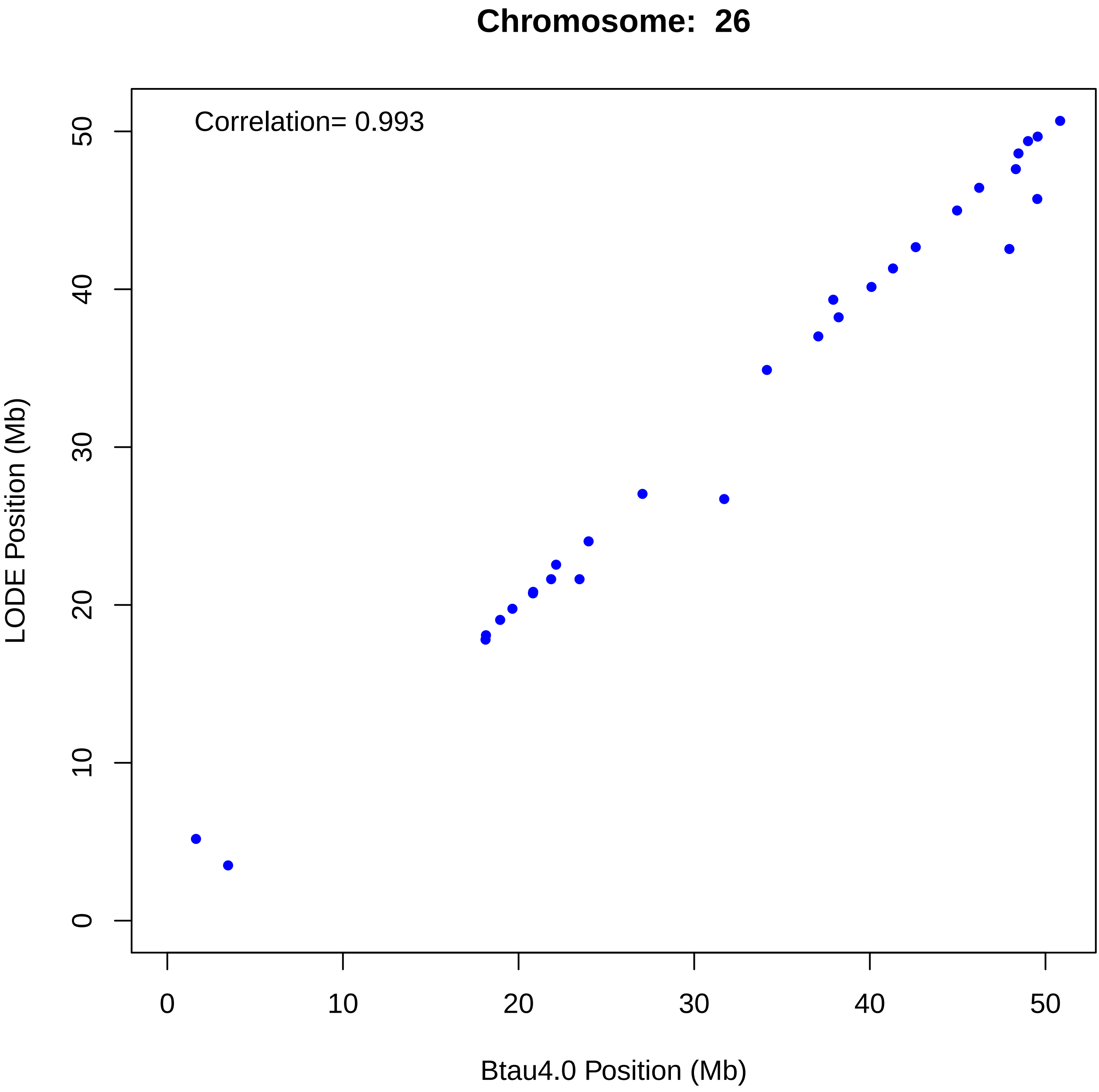

Chromosome: 26
Correlation= 0.993
LODE Position (Mb)
0
10
20
30
40
50
Btau4.0 Position (Mb)

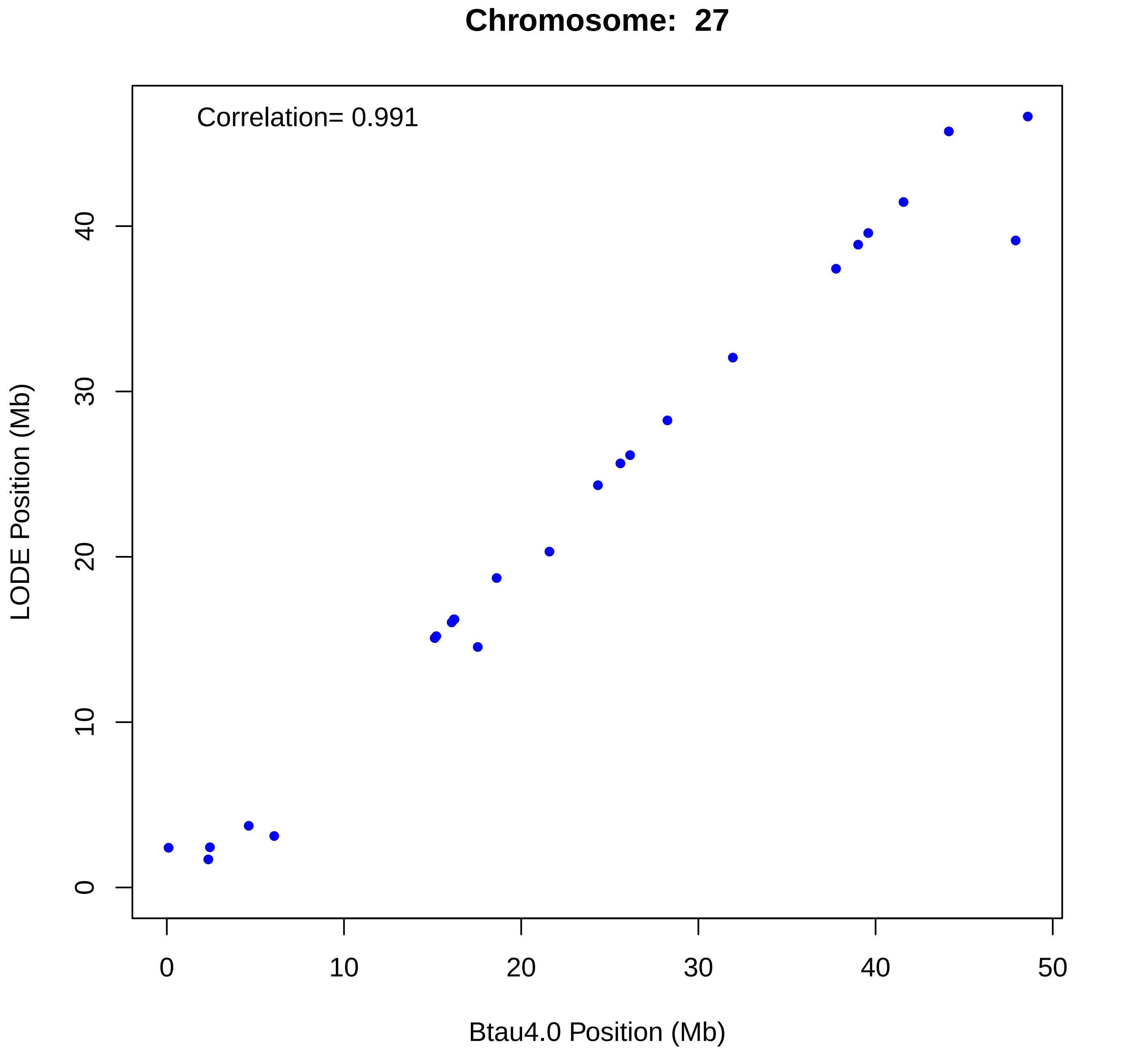

Chromosome: 27
Correlation= 0.991
LODE Position (Mb)
Btau4.0 Position (Mb)
0
10
20
30
40
50

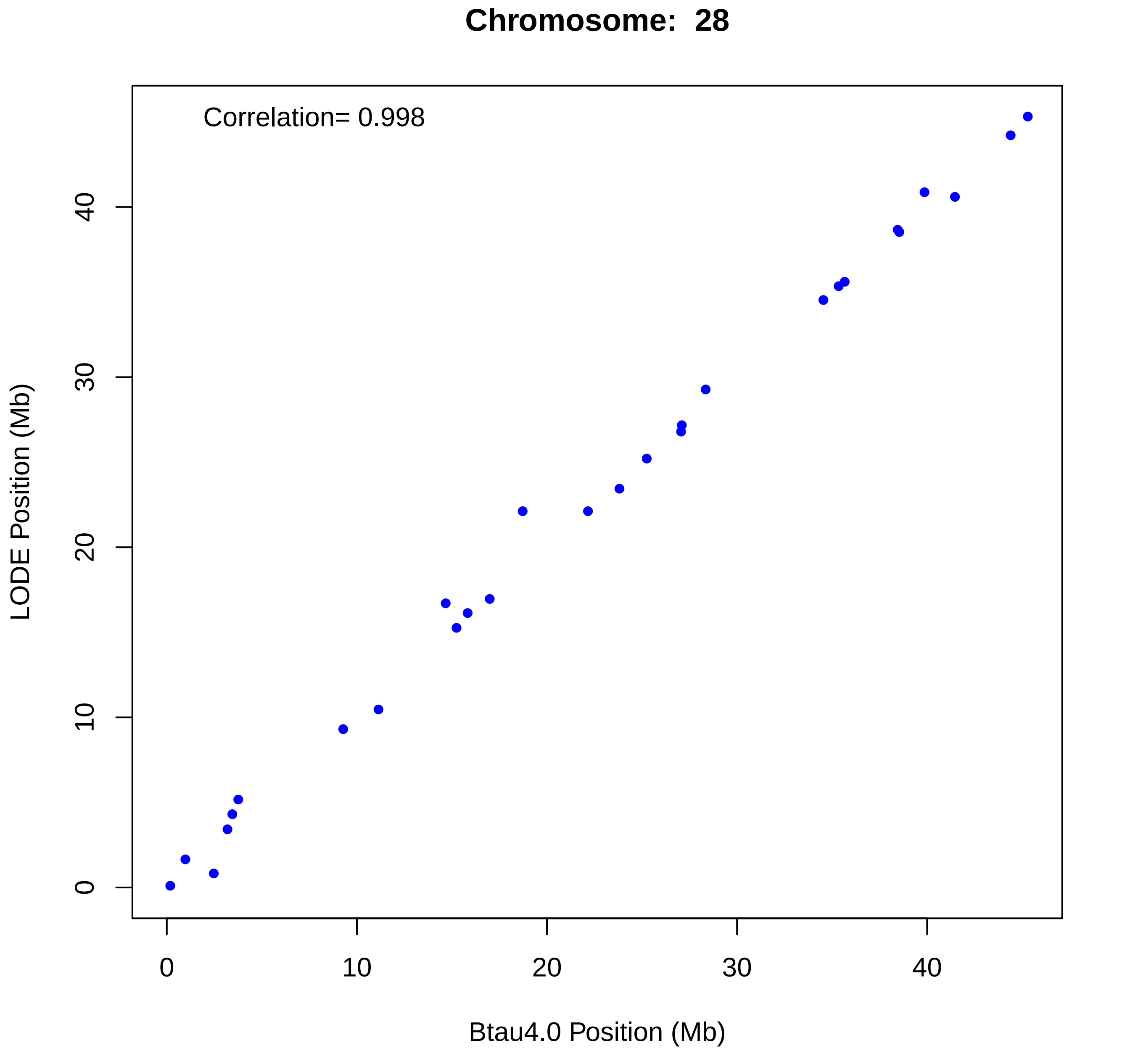
Chromosome: 28
Correlation= 0.998
LODE Position (Mb)
Btau4.0 Position (Mb)
0
10
20
30
40

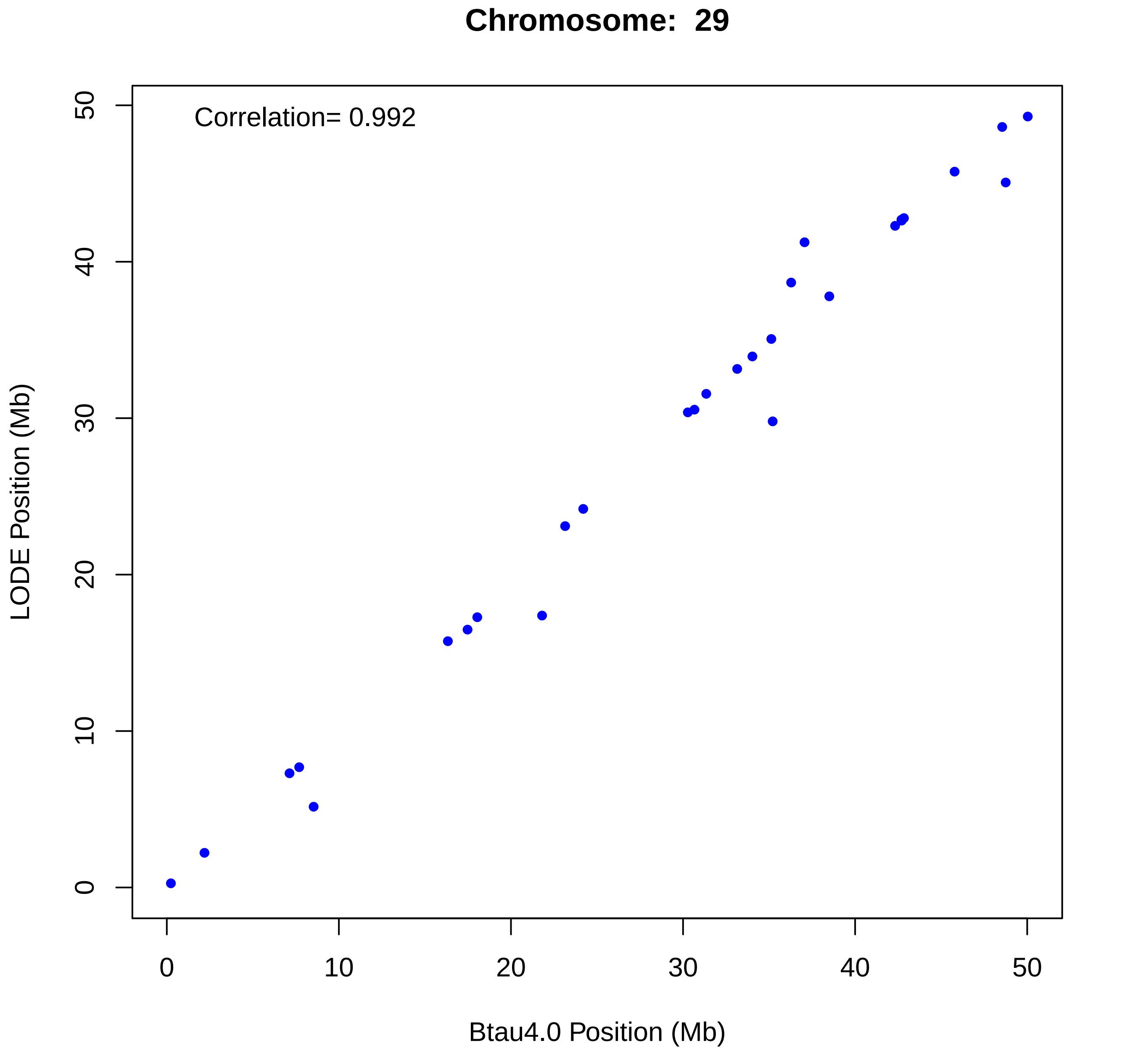
Chromosome: 29
Correlation= 0.992
LODE Position (Mb)
Btau4.0 Position (Mb)
0
10
20
30
40
50

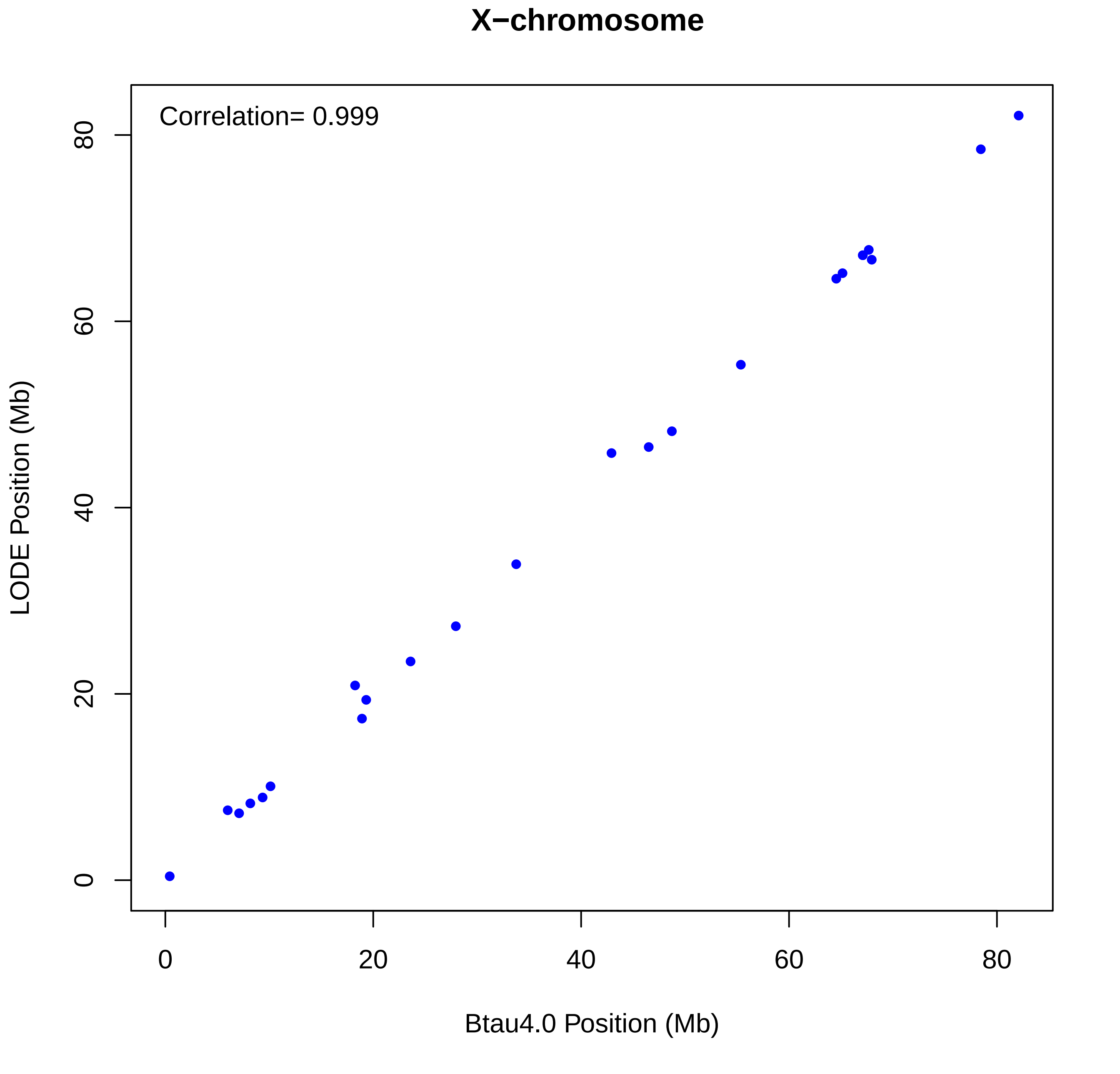
X−chromosome
Correlation= 0.999
LODE Position (Mb)
Btau4.0 Position (Mb)
0
20
40
60
80
