# Supplementary figures and images for: Assignment of chromosomal locations for unassigned SNPs/scaffolds based on pair-wise linkage disequilibrium estimates
Source: BMC Bioinformatics. 2010 Apr 7;11:171. doi: 10.1186/1471-2105-11-171 (PMC2859757; doi:10.1186/1471-2105-11-171)

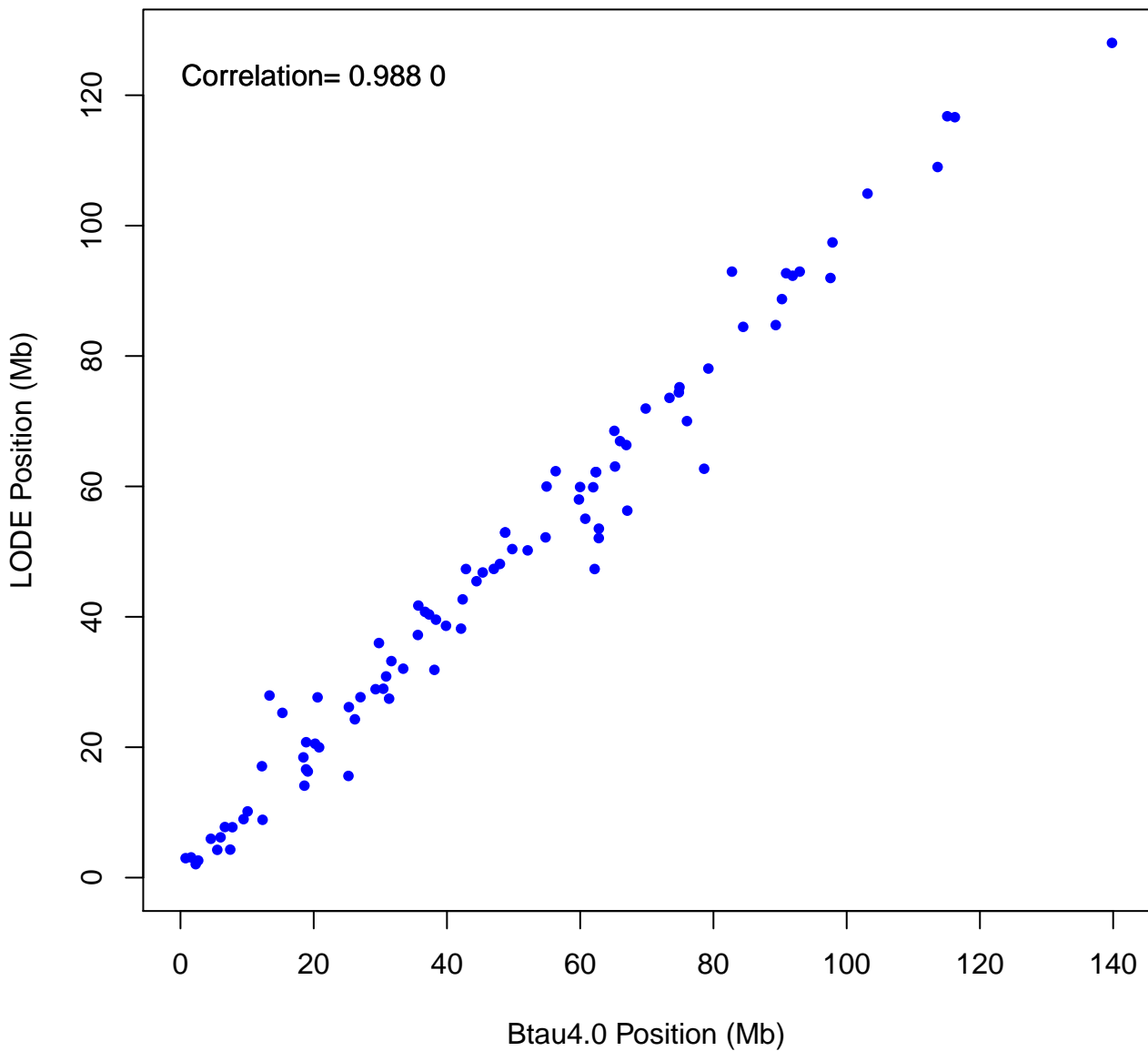

Supplement: Additional file 3 — The comparison of estimated (LODE) and known positions (Btau4.0) of 91 SNPs allocated a chromosomal position by LODE out a test set of 300 SNPs (0.01<MAF ≤ 0.05). The comparisons for positions are presented in a signal scatter plot for all the 91 SNPs combined over all chromosomes. [file 1471-2105-11-171-S3.PDF]
